# Enabling scalable inspection of offshore mooring systems using cost-effective autonomous underwater drones

**DOI:** 10.3389/frobt.2025.1655242

**Published:** 2025-10-16

**Authors:** Dong Trong Nguyen, Christian Lindahl Elseth, Jakob Rude Øvstaas, Nikolai Arntzen, Geir Hamre, Dag-Børre Lillestøl

**Affiliations:** 1 Department of Marine Technology, Norwegian University of Science and Technology, Trondheim, Norway; 2 Det Norske Veritas (DNV), Høvik, Norway

**Keywords:** autonomous underwater, drones, cost effective, aquaculture, maintenance and inspection, computer vision, path following

## Abstract

As aquaculture expands to meet global food demand, it remains dependent on manual, costly, infrequent, and high-risk operations due to reliance on high-end Remotely Operated Vehicles (ROVs). Scalable and autonomous systems are needed to enable safer and more efficient practices. This paper proposes a cost-effective autonomous inspection framework for the monitoring of mooring systems, a critical component ensuring structural integrity and regulatory compliance for both the aquaculture and floating offshore wind (FOW) sectors. The core contribution of this paper is a modular and scalable vision-based inspection pipeline built on the open-source Robot Operating System 2 (ROS 2) and implemented on a low-cost Blueye X3 underwater drone. The system integrates real-time image enhancement, YOLOv5-based object detection, and 4-DOF visual servoing for autonomous tracking of mooring lines. Additionally, the pipeline supports 3D reconstruction of the observed structure using tools such as ORB-SLAM3 and Meshroom, enabling future capabilities in change detection and defect identification. Validation results from simulation, dock and sea trials showed that the underwater drone can effective inspect of mooring system critical components with real-time processing on edge hardware. A cost estimation for the proposed approach showed a substantial reduction as compared with traditional ROV-based inspections. By increasing the Level of Autonomy (LoA) of off-the-shelf drones, this work provides (1) safer operations by replacing crew-dependent and costly operations that require a ROV and a mothership, (2) scalable monitoring and (3) regulatory-ready documentation. This offers a practical, cross-industry solution for sustainable offshore infrastructure management.

## 1 Introduction

**FIGURE 1:**
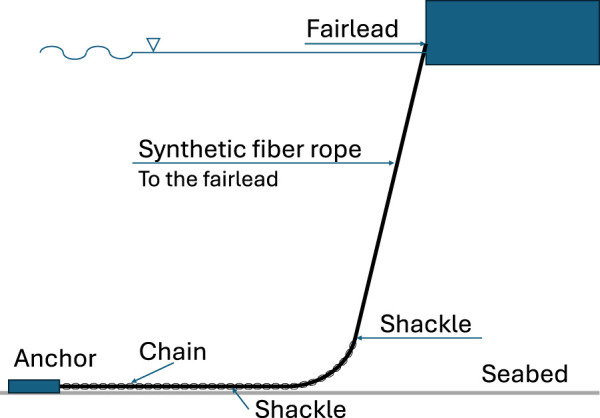
A simplified schematic of an offshore mooring system, with key components including line segments and connection components based on DNV-OS-E301 (Det Norske Veritas, 2024).

Marine-based industries such as aquaculture and floating offshore renewable energy (ORE) are undergoing rapid expansion and modernisation to meet rising global demands for food and clean energy (United Nations, 2023; International Energy Agency, 2024; Tiwari et al., 2012). The industry relies on mooring systems to maintain position, using anchored lines and connectors that must withstand harsh marine conditions. Figure 1 shows an example of catenary mooring system schematic including different segments and connection components (i.e., fairlead, shackle and anchor). These mooring lines degrade over time, e.g., fatigue, overload, corrosion, material degradation or mechanical damage (Kvitrud, 2014; Qiao, 2022; Bureau of Safety and Environmental Enforcement (BSEE) and ABS Consulting, 2015; ISO, 2019; Det Norske Veritas, 2015), so regular inspections are critical to ensure structural integrity (Det Norske Veritas, 2022). In practice, however, mooring inspections are infrequent–often only at scheduled intervals (annual or multi-year) – due to the high cost and complexity of current methods. This gap in coverage can allow failures to go unnoticed; indeed, there have been cases where a mooring line break remained undetected until the next periodic inspection (Bureau of Safety and Environmental Enforcement (BSEE) and ABS Consulting, 2015; Ford et al., 2020; Rahman et al., 2018). The consequences of an undetected mooring line failure can be severe, ranging from expensive downtime, economic loss of assets to even accidents, environmental catastrophes (Carpenter, 2015; United Nations, 2024; Labra et al., 2023; Yu et al., 2023). This highlights the need for more continuous and efficient monitoring.

Traditional mooring system inspection techniques are costly, labour-intensive, and not easily scalable (Subasinghe et al., 2009; Ford et al., 2020; Tait et al., 2023). Traditionally, divers or work-class ROVs (Remotely Operated Vehicles) are deployed to visually check mooring lines and hardware. Diver-based inspections, apart from exposing humans to risks, become exponentially more expensive and impractical in deep water environments (Bureau of Safety and Environmental Enforcement (BSEE) and ABS Consulting, 2015). ROV inspections improve safety by keeping divers out of danger, but they still require large support vessels and specialized operators, leading to high operational costs (Ford et al., 2020; Fun Sang Cepeda et al., 2023). These resource demands make it impractical to inspect a large number of mooring lines frequently or on demand. As a result, operators often limit inspections to infrequent surveys, which compromises scalability–the ability to cover many assets or to increase inspection frequency is constrained by manpower and budget. While the petroleum industry can tolerate high costs due to greater financial margins, aquaculture and renewable energy industries demand significantly leaner, cost-effective inspection solutions (DNV, 2024). This economic constraint highlights the need for scalable and affordable approaches to mooring inspections for the expanding number of installations (and hence the number of mooring lines) in these sectors.

Recent advances in marine robotics offer a pathway toward cost-effective and scalable inspection solutions. In particular, observation-class underwater drones, as classified by the International Marine Contractors Association (IMCA, 2024), are emerging as a promising tool for mooring line inspection. These vehicles are low-cost, portable, and easier to deploy compared to traditional work-class ROVs (Akram et al., 2022; Blueye Robotics, 2024). They can be launched from small vessels or platforms and operated by a minimal crew (Blueye Robotics, 2025), or can be a permanent residence in the sea (Skaugset et al., 2025), drastically reducing the logistics and expense per deployment. Modern observation-class ROVs can be equipped with monocular cameras, optional sonars, lights, a depth sensor, and Inertial Measurement Unit (IMU), enabling examinations of subsea structures. Researchers and early adopters report that such drones provide a viable solution, allowing more frequent and less costly underwater surveys without sacrificing coverage (Blueye Robotics, 2025). These smaller ROVs have the potential to increase inspection frequency while lowering costs, thereby improving the integrity management of mooring systems. Furthermore, with advancements in automation, these drones can be augmented with software for autonomous navigation and anomaly detection (Akram et al., 2022). This means that instead of a human manually piloting every inspection (which can be tiring and skill-intensive), the drone itself could follow a mooring line, gather footage/data, and flag potential issues, all with minimal human input. Such autonomy is key to enable scalability, as it would enable consistent inspections across many mooring lines and facilities without a proportional increase in labour.

Enabling autonomous mooring inspections with observation-class drones requires reliable perception and localisation methods. Recent techniques like camera-sonar fusion (Ludvigsen and Cardaillac, 2023) effectively combined visual detail and range detection but face challenges adapting to continuously changing mooring line angles (Hurtós et al., 2017). Acoustic communication was also suggested for underwater positioning (Garin et al., 2024). While Visual Simultaneous Localisation and Mapping (VSLAM) is promising (Zhang et al., 2022), real-time applications remain constrained by limited onboard processing of low-cost drones. IMU-based strategies (Santos et al., 2024) initially developed for floating offshore wind (FOW) platform’s structure could similarly benefit mooring inspections.

Today, manual review of lengthy video footage makes mooring inspection labour-intensive and subjective. VSLAM can offer autonomous, efficient inspection by enabling 3D reconstruction for automated defect detection (e.g., missing parts, marine growth, wear). Advances in monocular-inertial VSLAM from aerial robotics confirm feasibility in dynamic conditions (Alzugaray et al., 2017). Underwater applications using camera-sonar fusion (Hurtós et al., 2017) could face complexity and cost issues, making monocular VSLAM a more practical choice for mooring inspections.

Despite recent advancements, fully autonomous mooring line inspections using observation-class ROVs have yet to be realized in practice. Challenging underwater conditions, such as strong currents, dynamic obstacles, and low visibility (Guo et al., 2022), complicate the task of maintaining a drone’s proximity to mooring lines. Although regulatory standards like DNV-RU-OU-0300 (Det Norske Veritas, 2022) mandate General Visual Inspections (GVI) and Close Visual Inspections (CVI) to ensure structural integrity, inspections typically remain infrequent and Risk-Based Inspection (RBI) due to high operational costs.

A main research gap remains in enabling robust and efficient autonomous monitoring of mooring lines using observation-class underwater drones equipped with a minimum sensor package, including camera, IMU, and depth sensor. There is also limited research on how to leverage video data from such low-cost platforms to enable 3D reconstruction to support the defect detection over time. The research questions are then *“How can mooring lines be effectively monitored and inspected using cost-effective, off-the-shelf observation-class underwater drones, and how can the acquired visual data be used to perform 3D reconstruction for supporting the change detection and defect identification?”*.

The objective of this research is to develop a framework for autonomous mooring line inspection and monitoring, based on increasing the autonomy of affordable, off-the-shelf underwater drones equipped with basic visual and inertial sensors. This study focuses on the use of a commercially available low-cost underwater drone and leverages computer vision and object detection to enable autonomous navigation and inspection. VSLAM is used for 3D reconstruction of mooring lines to support change and defect detection. The framework is validated through simulation and sea-trial tests. The findings aim to serve as a foundation for developing standardised, scalable, and cost-effective solutions for mooring line inspection in both offshore aquaculture and ORE applications. The research builds upon and extends recent student work presented in Arntzen (2024) and Elseth and Øvstaas (2025).

This paper contributes with a modular and scalable approach that allows for an increased Level of Autonomy (LoA) while maintaining cost-effectiveness for inspection of mooring lines. By utilizing open-source tools such as Robot Operating System 2 (ROS 2) and commercial off-the-shelf (COTS) hardware, the inspection system is designed to be replicable, adaptable, and accessible, particularly for operators in emerging markets or smaller-scale operations. Moreover, this unified inspection approach is applicable to both aquaculture and offshore renewable energy systems, addressing the common challenge of mooring line inspection in these industries.

The remainder of this paper is structured as follows. In section 2, related work is thoroughly reviewed to identify gaps in current low-cost inspection technologies for underwater applications. Section 3 presents the environment and regulatory challenges associated with mooring line inspections. The proposed system architecture and its implementations are detailed in Section 4, including autonomy levels, sensor integration, simulation setup, and hardware platform. Sections 5, 6 outline the perception and control strategies, respectively. The experimental results from dock and sea trials are presented in Section 7, followed by discussions and conclusions in Sections 8, 9, respectively.

## 2 Literature review and research gap

This section provides an overview of relevant studies in underwater robotics, with a particular focus on technologies enabling low-cost autonomous inspection. The reviewed literature focuses on several key areas, i.e. (1) the use of Autonomous Underwater Vehicle (AUV), (2) ROS integration, (3) simulation frameworks using Gazebo, (4) VSLAM, (5) cost-efficient solution, (6) path-following strategies for applications such as mooring system inspections and related use cases, and (7) the target inspected object of mooring line (M) or others (O).

**FIGURE 2:**
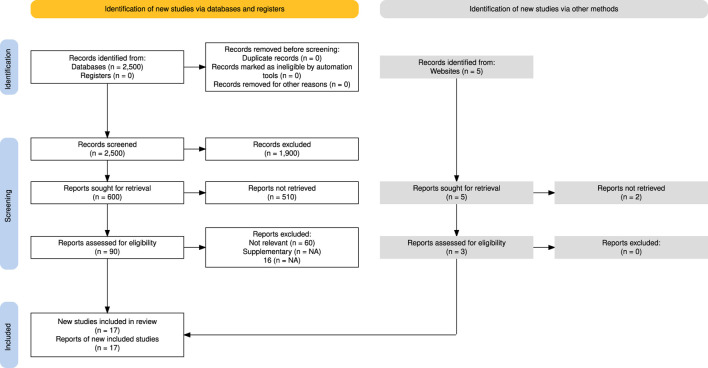
Study selection process using the PRISMA model (Haddaway et al., 2022, licensed under CC BY).

To ensure a systematic and transparent literature selection, the Preferred Reporting Items for Systematic reviews and Meta-Analyses (PRISMA) framework was applied (Haddaway et al., 2022). As shown in Figure 2, an initial pool of 2,500 articles was identified through searches in Web of Science, Semantic Scholar, and Scopus. After screening for duplicates and applying relevance criteria based on the seven (7) areas mentioned above, 22 studies were selected for detailed analysis. Further information on the search strategy is available in Elseth and Øvstaas (2025).

The selected literature is summarised in Table 1 where a checkmark indicates that a study explicitly addresses a given feature, and its relevance to mooring line inspection (the last column). A review of the matrix (Table 1) reveals that mooring line inspection remains underexplored, with most studies focusing on general underwater autonomy. Similarly, cost-effective solutions still need further research. Simulation using Gazebo and ROS are the next areas having room for more exploration. A horizon analysis of the matrix reveals that the studies from Manzanilla et al. (2019); Vithalani et al. (2020); Tipsuwan and Hoonsuwan (2015) align with several technical aspects of this paper in terms of cost-effective, autonomy, and path planning to follow a target object. However, they did not address mooring line inspection directly. Among few studies that did, only Maurelli et al. (2016) explicitly considered cost-effective solutions; however, it lacks integration of VSLAM and does not explore automatic defect detection using visual data from inspection campaigns.

**TABLE 1:** Relevant studies relating to cost-effective underwater drones.

References	Keyword	AUV	ROS	GAZEBO	VSLAM	Low-cost	Path	Application (M/O)*
Vargas et al. (2021)	BluROV/VSLAM	✓	✗	✗	✓	✗	✗	O
Guth et al. (2013)	Hippo VSLAM	✓	✓	✗	✓	✗	✗	O
Zhang et al. (2022)	Underwater SLAM	✗	✗	✗	✓	✗	✗	O
Shkurti et al. (2011)	State estimation	✓	✗	✗	✓	✗	✗	O
Manhães et al. (2016)	Gazebo ROV	✓	✓	✓	✗	✗	✗	O
Vithalani et al. (2020)	Navigation SLAM	✗	✓	✓	✓	✓	✓	O
Manzanilla et al. (2019)	ROV VSLAM nav	✓	✓	✓	✓	✓	✗	O
Zhao et al. (2022)	Mooring ROV	✓	✗	✓	✗	✗	✗	M
Tipsuwan and Hoonsuwan, (2015)	Pipeline Inspection	✓	✓	✓	✓	✗	✗	O
Xiang et al. (2010)	PI Multiple AUV’s	✓	✗	✗	✗	✗	✓	O
Zeng et al. (2015)	Long Range PI	✓	✗	✗	✗	✗	✓	O
Maurelli et al. (2016)	Chain following	✓	✓	✗	✗	✓	✓	M
Li et al. (2020)	VSLAM semantics	✗	✗	✗	✓	✗	✗	O
Yang et al. (2022)	Mooring	✗	✗	✗	✗	✗	✗	M
Willners et al. (2021)	Low-cost review	✓	✗	✗	✗	✓	✗	O
Allibert et al. (2019)	Girona-500 PI	✓	✗	✗	✗	✗	✓	O
Da Silva et al. (2022)	Aerial drone	✗	✓	✓	✗	✓	✓	O
Santos et al. (2024)	Dynamic path planning	✓	✗	✗	✗	✗	✓	O
Garin et al. (2024)	Tetherless positioning	✓	✗	✗	✗	✓	✓	O
Bremnes et al. (2024)	Risk modelling and path planning	✓	✗	✗	✗	✗	✓	O
Akram et al. (2025)	Net pen inspection	✗	✗	✗	✓	✓	✗	O
Grotli et al. (2016)	Autonomous job analysis	✓	✗	✗	✗	✗	✗	M

*M: Mooring system/O: other application.

### 2.1 Research inspiration and foundations

With the expansion of floating offshore infrastructure across the aquaculture and renewable energy sectors, the need for scalable and cost-effective underwater inspection solutions of mooring lines is becoming increasingly important. To enable routine inspections in scalable deployments, there is a need towards low-cost underwater autonomy through vision-based navigation and lightweight sensing. Nevertheless, the wide range of previous work within underwater robotics, VSLAM, low-cost autonomy, and inspection technologies need to be reviewed.

The feasibility of real-time underwater navigation using affordable platforms has already been demonstrated using monocular cameras and inertial sensors, for instance using a BlueROV2 from Blue Robotics equipped with basic onboard sensors (Blue Robotics, 2023). Even though these implementations did not include advanced depth estimation or full ROS integration, they showed the potential of vision-based control in low-cost systems and motivated further exploration into practical, deployable solutions (Manzanilla et al., 2019).

More advanced navigation and inspection capabilities have been achieved using high-resolution sensors such as sonar and multibeam imaging. These systems have enabled robust chain-following and localization in AUVs, albeit with potentially cost implications due to sonar sensors (Maurelli et al., 2016). While these results demonstrate the upper limits of inspection autonomy, their hardware requirements could limit adoption in cost-sensitive applications. Inspired by this, this paper aims to achieve similar levels of autonomy using only affordable visual sensors.

Valuable insights have also emerged from adjacent fields. In aerial robotics, hybrid approaches that combine deep learning and classical image processing have been explored, where convolutional neural networks (CNNs) and Canny edge detection were used to follow linear structures such as pipelines (da Silva et al., 2022). This combination of robustness and computational efficiency informed the architecture of the proposed system, which fuses You Only Look Once v5 (YOLOv5)-based object detection with Canny edge-based rope fitting.

As vision-based autonomy evolved, the importance of real-time localization and mapping became more evident. Monocular VSLAM systems such as Oriented FAST and Rotated BRIEF Simultaneous Localization and Mapping 2 (ORB-SLAM2) have been implemented in ROS environments to support real-time navigation and mapping (Vithalani et al., 2020). While this method was first designed for land-based robots, it has also been adapted for underwater use. Here, OctoMap is often used to create probabilistic occupancy grids when more advanced Simultaneous Localization and Mapping (SLAM) systems are not feasible (Arntzen, 2024). These tools enable low-cost spatial awareness within ROS-based systems, facilitating visual navigation in dynamic environments.

Simulation environments have played a crucial role in system development. The Gazebo simulator has been used for underwater drones, offering realistic force modelling and dynamic behaviour for control system development and validation (Manhães et al., 2016). While not focused on inspection tasks, such environments have been instrumental for prototyping visual guidance and autonomy logic before deployment in real-world conditions.

Recent advancements have also pointed toward future directions for autonomous inspection. For example, IMU-enhanced path planning has been applied to floating offshore platforms to improve navigation accuracy during inspection tasks (Santos et al., 2024). Similarly, acoustic-based localisation techniques have been proposed to enable tetherless operation when GPS fixes are unavailable (Garin et al., 2024). Approaches that incorporate risk-aware path planning have also been developed to support safer and more adaptive inspection strategies in uncertain offshore conditions (Bremnes et al., 2024). This state-of-the-art will help to increase the robustness of the underwater drones while increasing the LoA.

Altogether, these advancements point toward a convergence of cost efficiency and autonomy in underwater inspection. By building on developments in robotics, image processing, VSLAM, and simulation, this work aims to close the gap between expensive high-end systems and practical low-cost alternatives. The system presented here leverages commercially available hardware and open-source software to enable vision-based autonomous inspection of mooring lines, offering a scalable solution for both offshore renewable energy and aquaculture applications.

### 2.2 Current cost-effective design

In the aquaculture and ORE industry, the use of ROVs has shown a significant potential to reduce operational and maintenance (O&M) costs (Tait et al., 2023; Capocci et al., 2017) as well as providing high quality data (Khalid et al., 2022). Some typical applications of ROVs are inspection and maintenance of subsea and aquaculture infrastructure, environmental monitoring and deep sea mapping. Reflecting their diverse use cases, IMCA classifies unmanned underwater vehicles (UUVs) which are summarised in Table 2 for reference.

**TABLE 2:** Classification of UUVs.

Class	Description
Class I	Pure observation level
Class II, A	Observation level with load options
Class II, B	Observation level with mild investigation and intervention ability
Class III, A	Working level with weight around 1,000 kg and payload capability of 200 kg
Class III, B	Working level with weight around 3,000 kg and payload capability above 200 kg
Class IV, A	Towed underwater drone for cable laying
Class IV, B	For more accurate cable laying
Class V	Prototype or project-specific underwater drone
Class VI, A	AUV with weight less than 100 kg
Class VI, B	AUV weighing above 100 kg

In this paper, class I UUVs are of particular interest due to the availability of low-cost sensors such as a monocular camera, IMU, and depth sensors. Currently, Blueye Robotics is well established in the low-cost segment with drones such as the Blueye Pioneer and Blueye X3 (Blueye Robotics, 2024). At the time of writing this paper, the Blueye Pioneer has a lower price point ($5,554) compared to its successor, the Blueye X3, which is priced at $23,588. All Blueye Robotics drones are portable, user friendly, and come with open-source software. Although they are not autonomous out of the box, Blueye Robotics drones offer potential for implementing solutions that increase the LoA thanks to readily available Software Developer Kit (SDK).

Other cost-effective approaches to both underwater and aerial autonomy have been explored in recent studies. For instance, a COTS ROV is converted into an AUV in Willners et al. (2021). In this study the main focus was a BluROV2 (Blue Robotics, 2023), where the hardware and software challenges involved in transitioning from manual to autonomous operations were highlighted. Further, the potential of low-cost systems for broader adoption in underwater robotics is discussed.

Similarly, a vision-based method for autonomous pipeline inspection using a unmanned aerial vehicle (UAV) is proposed in da Silva et al. (2022). The paper utilised a standard PX4 flight controller integrated with ROS and simulated using Gazebo. A CNN was deployed to provide an initial estimate of the pipeline’s location, which was then improved using image processing techniques such as Canny Edge Detection for a more precise localization and path following of the pipeline.

### 2.3 Research gap in literature

While recent advancements in underwater robotics have demonstrated the potential of vision-based navigation and inspection, several key research gaps remain—particularly when it comes to creating cost-effective and scalable systems suited for real-world deployment. This paper addresses these gaps by exploring the integration of classical and deep learning-based perception, vision-only control strategies, and modular autonomy within a ROS 2-based architecture.

A major gap lies in the application of image processing techniques tailored to underwater environments. Few studies have examined how classical filtering approaches can suppress visual noise, such as marine snow, while retaining structural detail critical for reliable path following. Although such methods are computationally lightweight and compatible with resource-constrained hardware, their ability to generalize across lighting conditions and operational depths remains largely unexplored.

In parallel, deep learning models such as YOLO have shown success in terrestrial and aerial robotics, but their use for detecting structural elements like shackles or chain connections in subsea inspections is still in its infancy. The lack of annotated underwater datasets and the computational limitations of small-form-factor drones further hinder widespread adoption.

Another key area of limited research is the feasibility of vision-only control for underwater drones. Most documented systems rely on expensive navigation sensors such as Doppler Velocity Logs (DVLs) or multibeam sonar to ensure positioning and stability. While more affordable DVLs, like those offered by WaterLinked (2024), present promising alternatives, their integration in low-cost autonomous inspection systems remains largely untested.

Simultaneously, there is a clear lack of robust SLAM-based solutions for mooring line inspection. While SLAM techniques are well-established for general navigation, their application to underwater scenarios involving repetitive structures and depth-dependent lighting is rare. As shown in Table 1, only a handful of studies tackle these challenges. Moreover, SLAM frameworks such as ORB-SLAM3 are not yet fully adapted to ROS 2 environments, and available wrappers often lack critical features like real-time 3D point extraction (Haebeom Jung, 2023). Deploying such pipelines on platforms like the Blueye X3 is particularly difficult due to limited onboard computation and energy constraints.

Simulation is another underdeveloped aspect. Although Gazebo Garden provides next-generation capabilities for virtual testing, its integration with ROS 2 remains immature. For underwater systems, where real-world testing is costly, robust simulation environments are essential for validating control and perception pipelines before deployment.

Finally, while ROS 2 has become a widely adopted robotics middleware, the implementation of fully integrated, low-cost, ROS 2-based pipelines for underwater inspection is still rare. Community-developed tools such as yolov5_ros (Ar-Ray, 2024) and ROS 2-compatible ORB-SLAM3 wrappers (Haebeom Jung, 2023) offer a technical foundation, but there is limited evidence of these components being brought together into coherent systems for autonomous operation in real marine environments.

In summary, this paper responds to these gaps by proposing a vision-based, modular inspection system that relies solely on affordable sensors and operates without external positioning. It contributes to the field through the development of robust image processing pipelines, integration of deep learning-based detection, and validation of visual-only control through both simulation and sea trials.

## 3 Regulatory and environmental challenges

### 3.1 Mooring systems

Mooring systems are critical for maintaining the position of floating offshore structures such as wind turbines, fish farms, and FPSOs. Depending on site conditions, these systems may be taut, catenary, or tension-leg configurations (Rui et al., 2024; Wang, 2022a; b), each with specific challenges for autonomous inspection, especially across varying environments like midwater and seabed zones. Failures due to fatigue, overload, corrosion, material degradation or mechanical damage are well documented (Kvitrud, 2014; Qiao, 2022; Bureau of Safety and Environmental Enforcement (BSEE) and ABS Consulting, 2015; ISO, 2019; Det Norske Veritas, 2015) and can lead to severe operational and environmental consequences (Carpenter, 2015). This underlines the importance of regular and reliable inspection. A more detailed account of mooring system properties, configurations, and failure modes is available in the corresponding master’s theses (Arntzen, 2024; Elseth and Øvstaas, 2025).

### 3.2 Rules and regulations

To ensure the station-keeping and operability of moored floating structures, several rules are defined by Det Norske Veritas (DNV). *DNV-RU-OU-0300* defines in-service inspection regimes for FOW (Det Norske Veritas, 2021). Specifically, this standard sets requirements for annual interim surveys and a complete survey. The complete survey must be conducted within a 5 year interval and has different requirements dependent on the site-specific fatigue design life factor. Some relevant requirements include being a GVI of all mooring lines with comparison of video data from previous inspection campaigns, and a CVI of one mooring line from each mooring line cluster.

### 3.3 Properties of autonomous mooring line inspection using drones

Autonomous underwater inspection of mooring systems requires specific functional properties and system capabilities due to the geometric, environmental, and operational characteristics of the task. This section outlines the key requirements that inform the design of a vision-based, low-cost inspection drone system.

#### 3.3.1 Mooring characteristics

Mooring lines extend from the fairlead at the floating structure to an anchor on the seabed, covering a trajectory that may shift from near-vertical to horizontal. In addition, a mooring line experiences dynamics due to platform motion, hydrodynamic forces, and line elasticity. The inspection system must be capable of following this continuous span—often tens to hundreds of metres in length—while maintaining a stable trajectory and viewing angle. This requires a navigation strategy that supports line-following over varying orientations and depths.

#### 3.3.2 Underwater visibility

For camera vision in underwater robotics, one of the dominant challenges is the presence of marine snow, especially in deeper waters where no sunlight is present. Marine snow is a somewhat loosely defined term, but can be summarised as the presence of particles of different dimensions and transparency. The particles mainly consist of organic matter from zooplankton remains, fecal materials, and suspended sediments (Guo et al., 2022). Marine snow tends to move towards the seabed, but can also move in other directions depending on currents and the relative movement of the drone. Another factor is the brightness emitted from the light onboard the drone. Analogous to driving a car in the darkness with headlights in snow or rain, increased lighting causes the particles to appear more prominent.

Various methods are available for removing marine snow, with filtering techniques like median blur and deep neural networks being the most common (Jiang et al., 2020). However, neural networks demand substantial computational resources, making them unsuitable for low-cost drones with limited onboard processing capabilities. Cardaillac and Ludvigsen (2022) introduced an image enhancement technique that successfully removed the majority of marine snow present in the frame. This approach was promising as it allowed for real-time processing of video data before applying camera vision techniques.

Another challenge is the variable illumination conditions encountered at different depths. Near the surface, sunlight creates strong gradients and overexposed regions in the upper part of the image, while at higher depths, the scene is predominantly illuminated by the onboard LED, resulting in uneven lighting. These variations complicate consistent feature extraction and object detection across the inspection path.

#### 3.3.3 3D reconstruction of mooring lines for change and defect detection

While change detection (Adam et al., 2022), comparing historical and current inspection data, can support automated identification of structural degradation or anomalies (e.g., wear, damage, or missing components), today common practices still rely heavily on manual video review. AI-based methods have shown promise in reducing manual effort and improving accuracy, but their application in the inspection and maintenance of floating underwater structures remains limited, with only a few studies, exemplified by marine growth detection (Palla, 2024) or changes in risk profiles (Bremnes et al., 2024).

A first step for change detection is the ability to reconstruct the mooring line in 3D over time. However, this task is complicated by the inherent difficulties of the underwater environment, such as large data volumes, the dynamic nature of mooring lines, poor visibility, sensor noise, biofouling as well as the scarcity of well-labeled datasets. Unlike applications such as coral reef monitoring, which benefit from static, texture-rich scenes, mooring line inspection must contend with moving targets and feature-poor backgrounds. This paper addresses these challenges by focusing on 3D reconstruction using low-cost visual sensors, laying the foundation for future automated change and defect detection in complex underwater settings.

## 4 Proposed system architecture and implementation

### 4.1 Increased autonomy levels in COTS underwater drones

Defining autonomous control systems and distinguishing between an automatic and autonomous control system is not an easy task. In this paper, autonomy of a control system is defined as its ability to perceive an environment through sensors, process the information, and make context-appropriate decisions, and then act upon those decisions, all while adapting to familiar and unfamiliar conditions without human intervention. Autonomy is commonly characterised by levels. Currently, there is no internationally renowned taxonomy for the LoA applied to maritime robotics. In this paper, an adapted taxonomy from One Sea Ecosystem (2022) was used and summarised in Table 3.

**TABLE 3:** LoA in maritime robotics.

Level	Description
0 – Manual Operation	No autonomy; full human control
1 – Assistance	Supports tasks; constant supervision required
2 – Partial Autonomy	Some autonomy; still needs operator input
3 – Conditional Autonomy	Autonomous in known settings; human fallback
4 – High Autonomy	Fully autonomous in specific missions
5 – Full Autonomy	Self-sufficiency across all situations

COTS low-cost ROVs such as the Blueye X3 and BlueROV2 operate at Level 0 - Manual Operation, as outlined in Table 3. One could also argue that they operate at Level 1- Assistance given their built-in capabilities of maintaining heading and depth without human intervention. However, the drones can also be interpreted as ROVs, and several control and perception systems must be implemented before approaching a higher LoA. The developments presented in this paper aim to increase the LoA to Level 3 - Conditional Autonomy, wherein the drone can operate autonomously in a defined set of conditions, but may opt to human control if uncertain conditions are met. Although existing infrastructure has been demonstrated to support residential UUV autonomy (NTNU, 2025), achieving full autonomy for low-cost drones will necessitate further enhancements to enable reliable operation across a wider range of environmental conditions without human supervision.

Applying autonomy in maritime robotics is a tedious process. The primary challenge is the lack of GPS. In contrast to aerial applications of drones, GNSS sensor data is not available. Solutions do exist; for example, baseline (BL) acoustic positioning systems provide accurate positioning. However, such systems are not considered low-cost and are therefore not suited for cost-sensitive applications.

The scope of this paper is limited to vision-based autonomy without reliance on external positioning systems. First of all, unlike in aerial drone applications, underwater navigation lacks reliable GPS. Secondly, while accurate alternatives such as baseline acoustic positioning systems, they are not cost-effective and therefore fall outside the low-cost focus of this study. Similarly, sonar, with the capacity to fuse with cameras, is excluded in this paper due to additional payload and complexity it introduces. One may argue that acoustic and sonar technology could increase even further the Level of Autonomy (LoA), their integration is beyond the scope of this paper.

### 4.2 Hardware platform

Two promising low-cost COTS drones have been considered in this paper. The most affordable among them is the BlueROV2 from Blue Robotics (2023), known for its open-source electronics and movement in six degrees-of-freedom (6-DOF). López-Barajas et al. (2024) demonstrated the BlueROV2’s capability for aquaculture inspections, using deep learning and YOLO object detection to detect holes in fish cage nets. However, Blueye Robotics X3 ROV (Blueye Robotics, 2024) was chosen for this study due to its availability at the authors’ institution. In addition, an agreement with the manufacturer provides necessary support. A comparison of key parameters for both platforms is presented in Table 4.

**TABLE 4:** Comparison of BlueROV2 and blueye X3 specifications.

Specification	BlueROV2	Blueye X3
Dimensions (L × W × H)	457 × 338 × 254 mm	485 × 257 × 354 mm
Weight in Air	11–12 kg (with ballast and battery)	8.6 kg (with saltwater ballast)
Depth Rating	300 m	305 m
Forward Speed	1.5 m/s (3 knots)	1.5 m/s (3 knots)
Thrusters	6 (4 vectored, 2 vertical)	4 × 350 W
Battery Runtime	2–4 h	Up to 5 h
Camera Resolution	1080p, 110° FOV, ± 90° tilt	1080p, 115° vertical FOV, ± 30° tilt
Lighting	2 or 4 × 1,500 lumens, 135° beam	3,300 lumens, 5,000 K, CRI 90
Estimated price	4,600 USD	23,588 USD

The Blueye X3 is equipped with four available thrusters, allowing for translational movement in surge, sway and heave, as well as rotational movement in yaw. The Blueye X3 has a tether, allowing real-time video transmission to the remote control console and serving as a fail-safe in the event of thruster failure or battery depletion. Although the tether can be removed, it remained kept attached during trials for safety reasons. The drone will be subject to tetherless inspection at a later stage, once a sufficient LoA is achieved.

An additional advantage of the Blueye X3 is the availability of a Python SDK, which provides easy access to telemetry data and sending of thruster commands to the drone. The Blueye X3 also features three guest ports that support peripheral equipment such as acoustic sensors, cameras and grippers. Although guest ports are not utilised in this paper, they represent a desirable property that could support further advances of autonomy.

### 4.3 Overview of the system architecture

**FIGURE 3:**
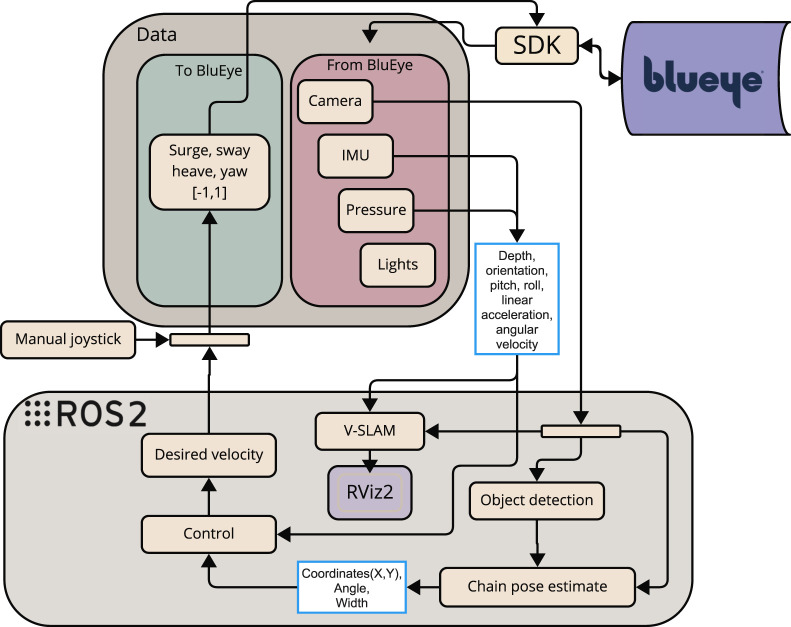
Proposed system architecture for Blueye X3 (Arntzen, 2024).

The architecture of the system is designed around a modular perception-control loop, as illustrated in Figure 3. The system starts with the camera input from the ROV, which acts as the primary sensor for visual data collection. This data is sent to a pre-processing node, where classical computer vision techniques such as Guided Filtering, Contrast Limited Adaptive Histogram Equalization (CLAHE), and morphological operations are applied to improve image quality and noise filtering, including marine snow.

Further, the visual stream is divided into two parallel processing pipelines. One pipeline leads to an *Object detection* node (in Figure 3) driven by YOLOv5, which is trained to identify and mark shackles found on the mooring line. The other pipeline proceeds to a mooring line detection module (lower right corner of Figure 3), which extracts geometric features of the mooring line.

The outputs from both pipelines are fused in a unified perception node. This module generates a message containing both the visual tracking state of the mooring line and detection flags from the object detection model. This message is published as a YoloCannyChainPose and passed to the decision-making node, which interprets the incoming visual data to determine actions.

Finally, the *Control* node (in Figure 3) translates these decisions into actuator commands in surge, sway, heave, and yaw which are then transmitted to the Blueye X3. The actuator commands are sent through the DesiredVelocity topic to the thrust allocation system onboard the drone.

It is noted that the *VSLAM* node in Figure 3 will be performed offline on recorded videos. This will be presented later in subsection 5.3.

#### 4.3.1 Calibration

Sensor calibration is important to ensure accurate operation of drone’s positioning. Camera calibration is necessary to correct for lens distortion and skewness, while IMU calibration enables estimation of key noise parameters such as white noise and random walk. The camera and IMU calibration parameters are listed in Tables 5, 6.

**TABLE 5:** Camera calibration parameters in air and underwater (Arntzen, 2024).

Parameter	Air	Underwater
Camera Type	Pinhole	Pinhole
$f_{x}$	987.628	1,203.945
$f_{y}$	998.105	1,202.857
$c_{x}$	955.953	977.854
$c_{y}$	529.845	537.217
$k_{1}$	−0.216	−0.167
$k_{2}$	0.0483	0.0396
$p_{1}$	0.000816	0.002709
$p_{2}$	0.000444	0.004614
$k_{3}$	0.0	0.0
Image Width	1920	1920
Image Height	1,080	1,080

**TABLE 6:** IMU noise model parameters (Arntzen, 2024).

Parameter	Value	Units
Gyroscope “white noise”	1.698e-04	$\frac{\text{rad}}{\text{s}\sqrt{\text{Hz}}}$
Accelerometer “white noise”	2.0e-03	$\frac{\text{m}}{{\text{s}}^{2}\sqrt{\text{Hz}}}$
Gyroscope “random walk”	1.939e-05	$\frac{\text{rad}}{{\text{s}}^{2}\sqrt{\text{Hz}}}$
Accelerometer “random walk”	3.0e-03	$\frac{\text{m}}{{\text{s}}^{3}\sqrt{\text{Hz}}}$
IMU sampling rate	1.0e3	Hz

**FIGURE 4:**
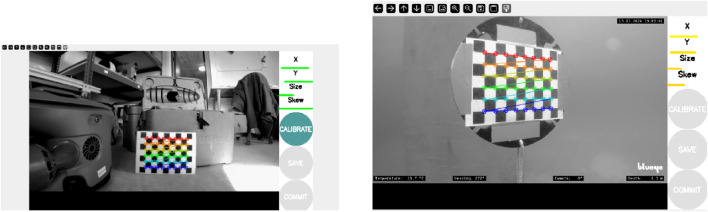
Camera calibration performed in air (left) and underwater conditions (right) (Arntzen, 2024).

The camera was calibrated both in air and underwater using the ROS 2 camera calibration package. The calibration was performed with a 6×8 checkerboard pattern at 30 frames per second (fps), using a native ROS 2 command. The resulting parameters are listed in Table 5, and the calibration images are shown in Figure 4.

The IMU calibration was performed using the kalibr toolbox, where the Blueye X3’s onboard MEMS-based IMU was calibrated using AprilTags and a recorded ROS bag. The final noise model parameters are listed in Table 6.

#### 4.3.2 Drone sensors

The COTS Blueye X3 is equipped with internal sensors that publish data to specific ROS 2 topics, which can subsequently be subscribed to by other system components. These topics store data such as thruster forces, pose, orientation, and video from the onboard camera. Pose and orientation are gathered from an IMU sensor. The IMU gathers data from a gyroscope, an accelerometer, and a magnetometer. The IMU data indicates the relative pose and orientation in comparison to an earlier reference frame or initial state.

Depth and orientation data are handled by the BluEye_Pose node, which publishes a pose message on the/BlueyePose topic. This message contains roll, pitch, and yaw data in addition to depth. Inertial motion data is provided by the IMU_to_ros2 node, which streams accelerometer, gyroscope, and magnetometer data to ROS topics such as/blueye/imu.

Moreover, to monitor the force set points from the drone in the 4-DOF, the BluEye_Force node reads thrust information in the surge, sway, and heave directions, and publishes it to/BlueyeForces. For visual feedback, the Video_to_ros2 node publishes the camera stream to the topic/camera. This video stream is used both for visual inspection and as input to the mooring line detection pipeline.

Together, these nodes form the interface layer of the control system, and the relationship between the sensor nodes and the rest of the system is illustrated in Figure 3.

#### 4.3.3 ROS 2 integration

**FIGURE 5:**
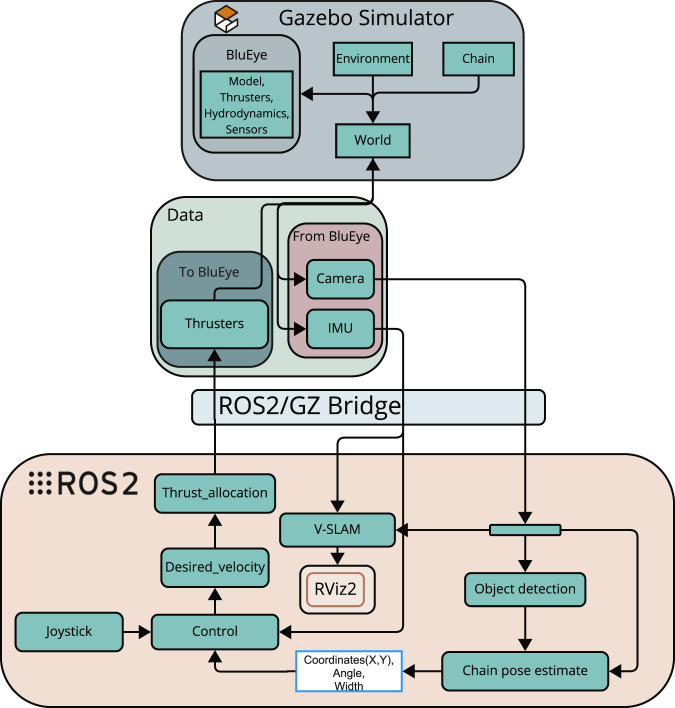
Simulated system architecture used for testing and validation (Arntzen, 2024).

The software architecture is implemented using ROS 2 (Macenski et al., 2022), which provides a modular node-based middleware for real-time message passing, node lifecycle management and topic-level Quality of Service (QoS) tuning. Perception nodes (image pre-processing, YOLOv5 detector, ORB-SLAM3 wrapper) publish visual state messages that are consumed by the guidance and control nodes (Figure 3). For simulation, the ROS2/GZ Bridge is used to forward simulated sensor topics from Gazebo to ROS 2 (Figure 5). Full topic/message definitions and launch files are provided in Arntzen (2024); Elseth and Øvstaas (2025) and in the accompanying implementation repository.

### 4.4 Simulation platform

Virtual commissioning via simulation platforms enables early detection of problems, which can save both time and resources during real-life deployment. However, careful considerations must be made to ensure that the results from the simulated environment are replicable in a real-life scenario.

A simulator from the Applied Underwater Robotics Laboratory (AUR-Lab) (AUR-Lab, 2024), which is built on Gazebo Garden (AURLab, 2025), has been used in this work due to its capability to render a 3D environment with hydrodynamic and thruster forces. Observing Table 1, Gazebo Garden is not used in a previous work. In this paper, we will use the simulator framework developed by the AUR-Lab.

Gazebo Garden is a 3D dynamic simulator that works with ROS and a physics engine to develop and test robotic applications in a simulated environment. Gazebo is built on the physics engine Dynamic Animation and Robotics Toolkit (DART) which provides algorithms for the dynamics and kinematics of a robot’s movements in an environment. Gazebo Garden is the latest version of Gazebo which was released in 2022 (Gazebo, 2022).

Moreover Gazebo uses a modular architecture, and with its ROS integration, messages can automatically be converted between ROS and Gazebo. An explanation of how this is implemented in this project is discussed later in this section.

#### 4.4.1 Installation and system setup

To support development and testing of the autonomous inspection framework, a simulation environment was established using Ubuntu 22.04.5, ROS 2 Humble, and Gazebo Garden. Because Gazebo Garden is not yet fully supported on Windows platforms, a dual-boot configuration was required to ensure compatibility and performance. The system includes three principal components: Gazebo Garden for simulating the underwater environment, the ros_gz bridge for interfacing ROS 2 with the simulation, and a ROS 2 workspace containing all custom perception and control nodes.

#### 4.4.2 Simulated system architecture

Figure 5 provides an overview of simulated system architecture used for testing and validation. Within the Gazebo simulator, a virtual model of the Blueye operates in a 3D world that includes hydrodynamics, thruster modelling, and a mooring chain. Simulated sensor data, such as video from the onboard camera and inertial measurements from the IMU, are published through Gazebo and bridged into ROS 2. This data is used by the perception pipeline, which includes both object detection and mooring line tracking algorithms. The output of these nodes is fused into a pose estimate for the mooring line, which is published to the topic/ChainPos.

Parallel to this, a joystick node provides manual control inputs via the/joy topic. These inputs are parsed and merged with autonomous control outputs in the/blueye_joystick_parser node to form a desired velocity set-point. This set-point is then published to/blueye/desired_velocity and processed by the thrust allocation module. The resulting commands are distributed across the simulated thrusters and sent back to Gazebo using the following command:/model/blueye/joint/thruster_joint_ {1‥4}/cmd_thrust.

Throughout this process, the ros_gz_bridge maintains synchronization between the simulated world and the ROS 2 system. Visualization of the drone’s estimated motion and environment is handled through RViz2 (a visualisation tool for ROS 2), making effective testing of both perception and control in the loop possible.

#### 4.4.3 Image processing in simulation

**FIGURE 6:**
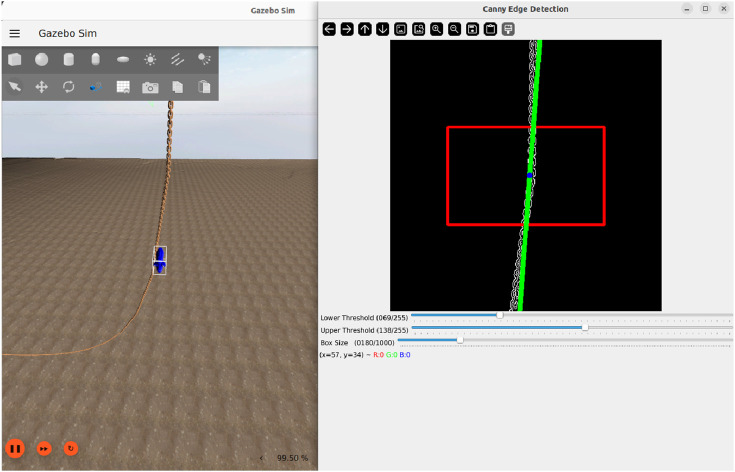
Visualization of the image processing pipeline in simulation. Left: Simulated environment. Right: Overlay with ROI (Region of Interest, red box) and fitted line representing the mooring line (green) (Elseth and Øvstaas, 2025).

The Gazebo simulator generates synthesis video data through a virtual camera, which is streamed in real time to the ROS 2-based control system (Figure 5). Figure 6 illustrates a sample frame from the Gazebo environment alongside the corresponding image processing output. In this example, the detected mooring line is overlaid in green, and the estimated mid-point is marked in blue. Relevant features (centre coordinates, orientation angle, line width, and frame brightness) are extracted and published to the controller for further processing. Image processing will be presented in the next Section 2.

## 5 Perception system

This section iteratively introduces the theory and corresponding implementation for the perception system which has been developed to increase the LoA of the Blueye X3.

### 5.1 Image processing

To enable mooring line detection using the Blueye X3’s camera, several image processing techniques were implemented, selected for their real-time performance and adaptability through parameter tuning.

#### 5.1.1 Theory

Colour Space Conversion converts the pixels found in the video frames from the Blueye camera from Red, Green, Blue (RGB) to a YCbCr format. In this space, the image is represented by a luminance component (Y) and two chrominance components ( Cb and Cr ), which encode colour differences. Each of the three RGB values is represented with an intensity value in the range 0–255. Marine snow appears as white and gray spots in the frame. This visual characteristic suggests that most of the relevant image information for marine snow is encapsulated in the luminance component Y of the YCbCr colour space (Cardaillac and Ludvigsen, 2022). To retain image features relevant to marine snow while reducing data dimensionality, the RGB colour space is converted to YCbCr. The RGB image can be represented as a vector, given by

$$I_{\mathrm{RGB}}\left(x,y\right)=\left[\begin{array}{c} R\left(x,y\right)\\ G\left(x,y\right)\\ B\left(x,y\right) \end{array}\right].$$ (1)

The RGB image (Equation 1) can then be converted to YCbCr colour space, given by

$$\begin{array}{ll} I_{\mathrm{Y CbCr}}\left(x,y\right) & =\left[\begin{array}{ccc} 0.299 & 0.587 & 0.114\\ -0.168736 & -0.331264 & 0.5\\ 0.5 & -0.41688 & -0.081312 \end{array}\right]\\ & \;\cdot \left[\begin{array}{c} R\left(x,y\right)\\ G\left(x,y\right)\\ B\left(x,y\right) \end{array}\right]+\left[\begin{array}{c} 0\\ 128\\ 128 \end{array}\right]. \end{array}$$ (2)

For the subsequent steps in the image processing pipeline, only the luminance component, e.g., Equation 2, of each video frame is used.

Guided Filtering is applied to smooth out the image and reduce the noise from marine snow. First, given a guidance image, its intensity value I(i,j) of each pixel (i,j) is found. For each pixel, neighborhood pixels are selected with a window radius r containing ω pixels. The mean, correlation and variance of each pixel I and its neighborhood are calculated as

$$\bar{I}\left(x,y\right)=\frac{1}{\left|\omega \right|}\underset{\left(i,j\right)\in \omega \left(x,y\right)}{\sum }I\left(i,j\right),$$ (3)

$$I_{\text{corr}}\left(x,y\right)=\frac{1}{\left|\omega \right|}\underset{\left(i,j\right)\in \omega \left(x,y\right)}{\sum }I{\left(i,j\right)}^{2},$$ (4)

$$I_{\text{var}}=I_{\text{corr}}-{\bar{I}}^{2},$$ (5)

where I is the pixel intensity value at coordinates (i,j) ; $\bar{I}\left(x,y\right)$ is the local average intensity within window ω(x,y) of radius r , serving as a baseline for smoothing; $I_{\text{corr}}\left(x,y\right)$ is the local correlation; $I_{\text{var}}$ is the variance, i.e., high variance indicates edges, while low variance corresponds to smooth regions. Secondly, the three constants a , b and q are calculated as

$$a=\frac{I_{\text{var}}}{I_{\text{var}}+\epsilon }$$ (6)

$$b=\frac{\bar{I}}{1-a}$$ (7)

$$q=\bar{a}*I+\bar{b},$$ (8)

where ϵ is a regularization term which balances between edge preserving and smoothing. The mean values of a and b are calculated with the same equations used for finding $\bar{I}$ . Finally, the filtered output of the image q is calculated. The coefficient a controls the trade-off between smoothing and edge preservation (with a≈1 at edges and a≈0 in homogeneous regions); b adjusts the local intensity offset to maintain the neighborhood mean; and the output q combines these terms in a linear model to produce the final edge-preserving smoothed image. More details can be found in He et al. (2013).

Contrast Limited Adaptive Histogram Equalization (CLAHE) is applied to improve the contrast in the image. In this method, the pixel intensities in an image frame are visually represented in a histogram. CLAHE works by enhancing the contrast using Adaptive Histogram Equalization (AHE) (OpenCV, 2024b). In simpler terms, AHE stretches the histogram in order to improve the contrast of the image.

In this method, the image is divided into subsections called “tiles”. Each tile size is 8x8 as standard but is subject to tuning based on the desired output. For each tile in the image, AHE is applied to enhance the contrast. A major drawback with AHE is that it will introduce added noise to the image. To counter this, contrast limiting is applied to each tile. Contrast limiting ensures that the contrast of each individual tile does not surpass a set contrast limit. If a pixel is found to be above the set contrast level it is clipped and distributed evenly to other tiles within the image. The entire algorithm is described as follows, starting with histogram equalization at its core. Given a grayscale image with L possible intensity levels, the normalised histogram is defined as

$$p_{f}\left(i\right)=\frac{n_{i}}{n},\;0\leq i<L,$$ (9)

where $n_{i}$ is the number of pixels with intensity i , and n is the total number of pixels in the image. In this paper, L is set to 255, corresponding to the maximum intensity value of a pixel in an 8-bit image.

The cumulative distribution function (CDF) of the histogram is given by

$$T\left(i\right)=\overset{i}{\underset{j=0}{\sum }}p_{f}\left(j\right),$$ (10)

and is used to map each intensity level i to a new level $i^{\prime }$ according to

$$i^{\prime }=\lfloor T\left(i\right)\cdot \left(L-1\right)\rfloor .$$ (11)

The pixel values in the image are then updated using the transformation

$$f_{\text{eq}}\left(x,y\right)=\lfloor T\left(f\left(x,y\right)\right)\cdot \left(L-1\right)\rfloor ,$$ (12)

where $f_{\text{eq}}$ denotes the histogram-equalized version of the input image.

In the case of CLAHE, the image is first divided into non-overlapping tiles. A histogram is calculated for each tile, and the values are clipped at a predefined threshold, according to

$$H_{\text{clipped}}\left(i\right)=\min\left(H\left(i\right),T_{\text{clip}}\right),$$ (13)

where $T_{\text{clip}}$ is the clip limit. The clipped excess E is then redistributed uniformly across all histogram bins, according to

$$H_{\text{redistributed}}\left(i\right)=H_{\text{clipped}}\left(i\right)+\frac{E}{L}.$$ (14)

The local cumulative distribution function $T_{k}\left(i\right)$ for each tile k is computed as

$$T_{k}\left(i\right)=\overset{i}{\underset{j=0}{\sum }}p_{k}\left(j\right).$$ (15)

The remapped intensity is then given by

$$i_{k}^{\prime }=\lfloor T_{k}\left(i\right)\cdot \left(L-1\right)\rfloor .$$ (16)

Finally, bilinear interpolation is applied between adjacent tiles to avoid discontinuities, and the enhanced image is obtained as $f_{\text{CLAHE}}\left(x,y\right)$ .

After obtaining the contrast-enhanced image, a Morphological Transformation is applied to improve the quality of the image used for mooring line detection. Morphological operations are particularly useful for refining shapes in images, especially in noisy or low-contrast underwater scenes. In this case, erosion is used to clean up the image by removing small, irrelevant noise and isolating more prominent features. Erosion works by scanning the image with a small structuring element (also called a kernel), which can be shaped as a rectangle, ellipse, or cross. When the kernel passes over the image, each pixel is set to zero (i.e., background) if any of its neighbouring pixels within the kernel area are also zero. This results in sharper edges around foreground objects and suppresses small, isolated noise, which might otherwise interfere with line detection.

Mathematically, let f:E→R be the luminance component and b:B→R be the structuring function. The erosion operation is defined as

$$\left(f⊖b\right)\left(x\right)=\underset{y\in B}{\inf}\left[f\left(x+y\right)-b\left(y\right)\right],$$ (17)

where, inf denotes the infimum (greatest lower bound). This operation computes the minimum value of the image f in the neighborhood defined by B , adjusted by the structuring function b .

Canny Edge Detection (OpenCV, 2024a) is the last part of the pipeline, but equally important, to support a binary representation of structural edges with optimal noise immunity. A prerequisite to applying edge detection is reducing the amount of noise found in the image, and Canny Edge Detection does this by applying a Gaussian filter. Following, a Sobel kernel is applied horizontally and vertically to attain images with the first derivatives in horizontal $G_{x}$ and vertical $G_{y}$ directions. These two images are used to calculate the edge gradient G and direction θ , given by

$$G=\sqrt{G_{x}^{2}+G_{y}^{2}}$$ (18)

$$\theta_{G}=\tan^{-1}\frac{G_{y}}{G_{x}}$$ (19)

where $G,\theta_{G}$ are the edge strength and orientation at each pixel, respectively; and $G_{x},G_{y}$ are the horizontal and vertical gradients, respectively, calculated from Sobel kernels.

Subsequently, a non-maximum suppression is applied with a scan of the image in order to remove any pixels with no contribution to an edge. This is an iterative process where each pixel is checked with its neighbouring pixels in the vertical or horizontal plane. If the current pixel forms a local maximum compared to its neighbouring pixels, it is considered as an edge. Otherwise the pixel is suppressed and given a zero value. This scanning process is done horizontally, vertically and diagonally and is summarized in Algorithm 1.

Algorithm 1 Non-maximum suppression.

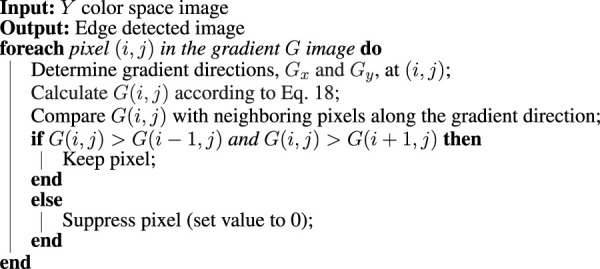

#### 5.1.2 Implementation

The proposed pipeline in this paper integrates multiple computer vision techniques as presented in Section 5.1.1) into a process, described in Algorithm 2. It is designed to balance performance and practicality, taking into account the computational limitations of the Blueye X3’s onboard hardware and the target frame rate of 30 FPS at 1080p resolution. By using OpenCV’s Python interface, the pipeline achieves both efficient real-time processing and development flexibility.

Algorithm 2 Image processing pipeline.

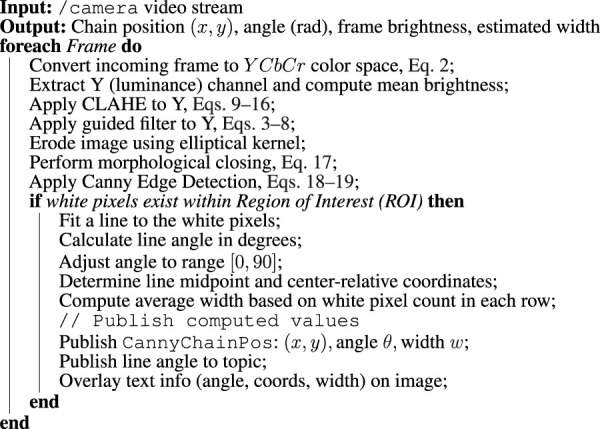

### 5.2 Object detection

A key part of a drone’s autonomy is its capability to detect different structural components on a mooring line. Detecting structural components is crucial for several reasons, with the major advantage being allowing for less use of manual human labor to detect structural changes on the mooring line. Another advantage, particularly relevant to this work, is the drone’s ability to switch from GVI to a CVI when approaching components on the mooring line that require detailed examination. Lastly, object detection can be used to determine the depth at which the AUV should initiate its ascent by detecting different components typically found when approaching the seabed.

#### 5.2.1 Theory

To detect different components, YOLOv5s, a lighter variant of YOLOv5 (Ultralytics, 2025), is used due to favorable properties such as real-time capability and high accuracy. In short terms, YOLOv5s works by using a pre-trained model, trained on real image data of mooring lines. This technique is called supervised learning, where the model is trained on a set of annotated data and then uses a defined set of algorithms to detect and classify components found in the image.

A major challenge with this method is the lack of sufficiently large annotated data. To address this, an annotation tool from (Roboflow, 2025) has been used. This allows for combined use of artificial intelligence (AI) and manual labeling to create annotated data which can be used with a broad range of object detection models.

YOLOv5s exhibits lower latency than its successors, making it a favorable choice for achieving full autonomy, where all computations have to be completed locally on the AUV. It is a convolutional neural network (CNN)–based object detector. The model works by processing each frame through an input layer which is then sent to a backbone network that extracts three hierarchical feature maps—P3, P4, and P5—corresponding to fine, intermediate, and coarse spatial resolutions. Each of these feature maps consist of different dimensions in the range of 20×20 to 80×80 pixels, depending on the input size. These feature maps are able to detect small, medium and large objects within the frame. After obtaining these feature maps, a confidence prediction and bounding box regression is executed to acquire a multi-dimensional array named *BBoxes*. This array contains essential information for each detected object such as object class, class confidence, normalised coordinates and dimensions. This process as a whole is referred to as an inference process (Liu et al., 2022).

YOLOv5s predicts bounding boxes relative to grid cells in a feature map. For each cell, it predicts four values: $s_{x},s_{y}$ ; normalised offsets for the box center within the cell, respectively; and $s_{w},s_{h}$ : normalised log-space scale values for width and height, respectively. These values must be transformed into actual positions of the bounding box in the input image space, given by

$$g_{x}=2\sigma \left(s_{x}\right)-0.5+r_{x},$$ (20)

$$g_{y}=2\sigma \left(s_{y}\right)-0.5+r_{y},$$ (21)

where $g_{x},g_{y}$ in Equations 20, 21 are the final absolute center position of the bounding box; σ is the sigmoid function, ensuring outputs are in (0,1), $s_{x},s_{y}$ are the raw outputs from the neural network, explained above; and $r_{x},r_{y}$ are the top-left corner coordinate of the current grid cell. The width and height of the bounding box are given by

$$g_{h}=p_{h}{\left(2\sigma \left(s_{h}\right)\right)}^{2},$$ (22)

$$g_{w}=p_{w}{\left(2\sigma \left(s_{w}\right)\right)}^{2},$$ (23)

where $g_{h},g_{w}$ in Equations 22, 23 are the final width and height of the bounding box; $s_{x},s_{y}$ are the raw predicted size offsets (learned by the network), as explained above; $p_{h},p_{w}$ are the anchor box dimensions (prior estimates for the box size in that grid cell).

To quantify the predictions of the model compared to ground truth values, a cost function is defined to include three components: classification, objectness, and localisation (Ultralytics, 2025), according to:

$$\text{Loss}=\lambda_{1}L_{\text{cls}}+\lambda_{2}L_{\text{obj}}+\lambda_{3}L_{\text{loc}}$$ (24)

where Loss in Equation 24 is the cost function value; $L_{\text{cls}}$ is the Classes Loss (or Binary Cross-Entropy loss) measuring the error for the classification task; $L_{\text{obj}}$ is the Objectness Loss (another Binary Cross-Entropy loss) penalising incorrect presence/absence predictions; $L_{\text{loc}}$ is the Location Loss (bounding-box) measuring the error in localizing the object within the grid cell; and $\lambda_{i}$ represents weights which can be subject to tuning. In this work, default hyperparameter values (Ultralytics, 2025) are used.

#### 5.2.2 Implementation

Object detection in the perception system is handled by a lightweight YOLOv5 model integrated into a ROS 2 wrapper node developed by Ar-Ray (2024). The model was trained to detect shackles which are fixed structures that in the case of mooring lines are used as a connection point between rope and chain. When a shackle is detected with confidence above a set threshold, the system flags this event and notifies the operator. The operator then makes a decision whether to initiate an autonomous ascent or continue descending. In this way, YOLOv5s is now used in a more targeted role for event detection rather than continuous tracking. This approach represents a human-in-the-loop strategy, and lays the foundation for higher LoA later.

**FIGURE 7:**
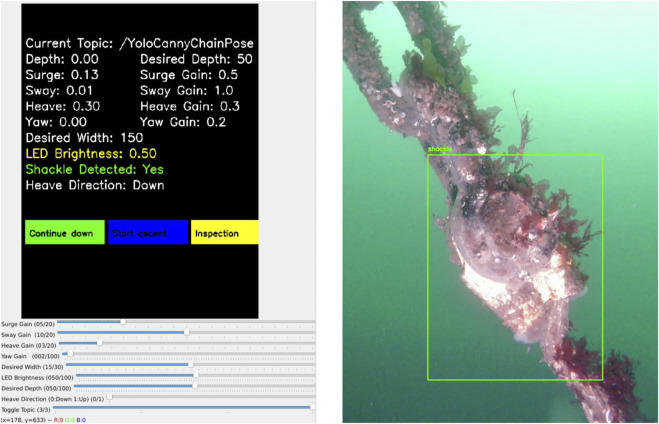
Left: System GUI with HIL prompt. Right: YOLOv5s bounding box around shackle.

On the left side of Figure 7, the graphical user interface (GUI) feedback during a successful shackle detection is shown. The perception system confirms a detection and prompts the operator to decide on the further mission. On the right side, the corresponding visual output from the trained YOLOv5s model is presented, highlighting the identified shackle.

### 5.3 Mapping and localization

#### 5.3.1 Theory

ORB-SLAM3 (Oriented FAST and Rotated BRIEF-SLAM3) is proposed as part of the 3D reconstruction of the mooring line structure (Arntzen, 2024). ORB-SLAM3 is an indirect feature-based SLAM framework suitable for real-time operation in challenging environments. Unlike direct methods, which are sensitive to lighting variability, ORB-SLAM3 uses Oriented FAST and Rotated BRIEF (ORB) OpenCV Team (2024) feature detection for robust pose estimation. However, it is important to note that ORB-SLAM3 has only been utilised for post-processing in this system, rather than for real-time operation.

The ORB algorithm is an open-source library built on the Features from Accelerated Segment Test (FAST) (OpenCV Team, 2018b) and BRIEF (OpenCV Team, 2018a). The FAST algorithm is a machine learning based approach that iterates through pixels in the image to determine if the pixel is a distinguishable feature in the image by examining the following criteria. Such a feature is typically a corner or an edge found in the image and is stored as a keypoint.

$$S_{p\to x}=\left\{\begin{array}{cc} d,\; & I_{p\to x}\leq I_{p}-t\;\left(\text{darker}\right)\\ s,\; & I_{p}-t<I_{p\to x}<I_{p}+t\;\left(\text{similar}\right)\\ b,\; & I_{p}+t\leq I_{p\to x}\;\left(\text{brighter}\right) \end{array}\right.$$ (25)

This is complemented with the BRIEF algorithm which iterates through the keypoints found from the FAST algorithm. BRIEF defines a binary vector τ based on the pixel intensity value corresponding to each keypoint that serves as numerical fingerprint which describes the area around each keypoint.

$$\tau \left(p;x,y\right)=\left\{\begin{array}{cc} 1\; & :p\left(x\right)<p\left(y\right)\\ 0\; & :p\left(x\right)\geq p\left(y\right) \end{array}\right.$$ (26)

**FIGURE 8:**
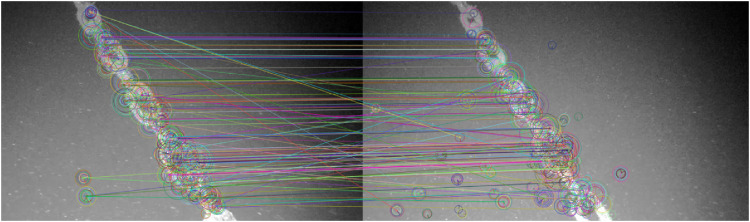
Feature matching from the sea trial in Arntzen (2024).

The ORB algorithm provides valuable information that can be used for loop closure by comparing each new frame in the video with previous keypoints found Loop closure is used to identify previously visited locations on a global map, and can reduce drift in system. A major challenge reported in Arntzen (2024) work is the repetitive structure of the mooring line. A mooring line is a homogeneous structure with few distinct features. Another challenge is the presence of marine snow. As shown in Figure 8, the system can to a certain extent successfully recognises features within the two frames, but struggles with some parts of the frames and also recognises particles as distinct features, which is not desired.

#### 5.3.2 Implementation

**FIGURE 9:**
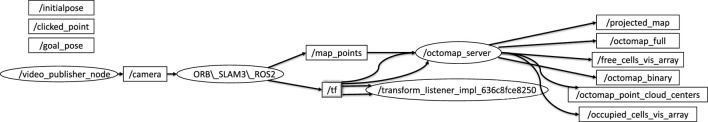
ORB-SLAM3 RQT graph (Arntzen, 2024).

Figure 9 illustrates how the ORB-SLAM3 is integrated into the system by Arntzen (2024). Input from a recorded video is published on the/camera topic by the/video_publisher_node, acting as the image source for the/ORB_SLAM3_ROS2 node. This node performs feature extraction and tracking, and publishes both the estimated map points and pose information.

The tracked 3D landmarks are published on the/map_points topic, while the robot’s estimated pose is shared via the/tf tree. To construct a map of the environment, the/map_points are forwarded to the/octomap_server, which builds an occupancy grid. The resulting map is then visualised through standard OctoMap topics.

## 6 Control system

The control system allows the drone to navigate along a mooring line using visual information extracted from the onboard camera. The control system is structured into three levels: (1) Planning and replanning, (2) Guidance, and (3) Control execution.

### 6.1 Planning and replanning

To ensure robustness, a fail-safe routine stops surge motion if no line is detected in a captured frame for 20 s. During this time, the drone slowly rotates in yaw in the direction the line was last seen to reacquire it.

Example 1 If the last recorded horizontal (x) coordinate of the fitted line is positive, indicating that the mooring line was last seen far right of the image, the yaw value is set slightly positive inducing a panning motion towards the most plausible position of the mooring line. The opposite logic would apply if the mooring line was last seen to the left of the image.

### 6.2 Guidance

**FIGURE 10:**
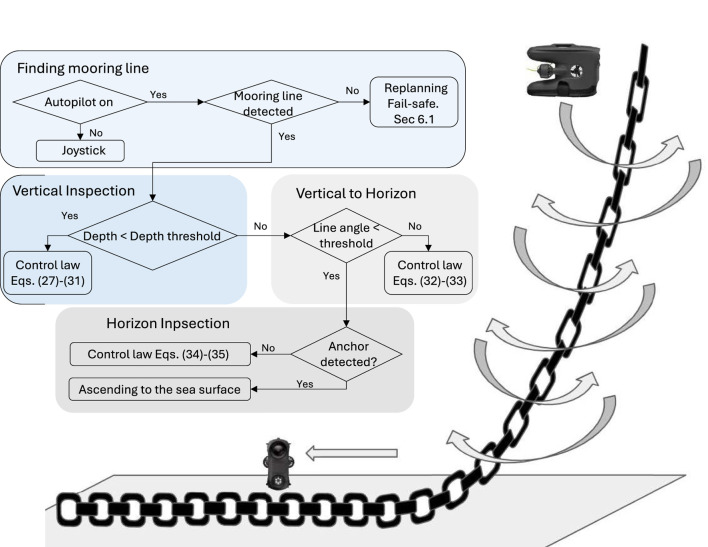
Control strategy flow chart (adopted from Arntzen (2024)). In the right hand side, the spiral pattern is shown.

The guidance system is structured into three stages: (1) Vertical Inspection, (2) Vertical-to-Horizontal Transition, and (3) Horizontal Inspection, as illustrated in Figure 10. Image processing provides real-time estimates of mooring line features. The object detection algorithm, trained on chain, rope, and wire mooring lines, can identify and process any combination of these types. Once a mooring line is detected in the video frame, the system extracts three key features (see Section 5.1): (1) the line’s width (in pixels), (2) the line’s midpoint position, and (3) the inclination angle. These measurements serve as a guidance for the drone to follow the mooring line, with the objective of maintaining a consistent distance from the line and keeping it centred in the camera view. This guidance is described by.

• Surge guidance is based on the perceived width of the mooring line, which acts as a proxy for distance.

• Sway and Yaw guidance is based on the midpoint horizon position of the mooring line in the captured frame. This guides the drone to right if the midpoint of the mooring line in the capture frame is on the right of the captured frame and *vice versa*.

• Heave guidance follows the inclination angle of the line such that it descends faster for a vertical incline angle and slower for a less vertical angle.

### 6.3 Control execution level

The control system ensures the drone follows the guidance system by regulating its motion in four degrees of freedom (DOF): surge, sway, heave, and yaw. Three control modes are defined based on the mooring line geometry: Vertical Inspection, Vertical-to-Horizontal Transition, and Horizontal Inspection, as illustrated in Figure 10.

#### 6.3.1 Vertical inspection

In this mode, the drone descends along a near-vertical mooring line. The control laws are defined as follows.

Surge regulates the distance to the mooring line, based on its width w in the image, according to a proportional controller:

$$\text{Surge: }F_{\text{surge}}^{\mathrm{vertical}}=\left\{\begin{array}{cc} k_{\text{surge}}\cdot \left(1-\frac{w}{w_{d}}\right),\; & \text{if }w\leq w_{d}\\ -k_{\text{surge}}\cdot \left(\frac{w-w_{d}}{200}\right),\; & \text{if }w>w_{d} \end{array}\right.$$ (27)

where $k_{\text{surge}}$ is the proportional gain to drive the width error to zero, ensuring the drone maintains the desired standoff distance; w is the observed line width in the captured frame; and $w_{d}$ is the target width.

Sway: two strategies are available. The first strategy (Sway1) regulates the drone on one side of the mooring line, given by

$$\text{Sway}1\text{: }F_{\text{sway}}^{\text{vertical}}=k_{\text{sway}}\cdot x_{\text{norm}},$$ (28)

where $k_{\text{sway}}$ is the proportional gain to keep the mooring line in the middle of the captured frame; $x_{\text{norm}}\in \left[-1,1\right]$ is the normalised horizontal offset of the line midpoint in the captured frame. The second strategy (Sway2) regulates the drone in a “spiral” pattern by alternating the sway controlled force between two compass points or by using a timer. This control methodology creates an alternating semi-helical pattern down the vertical section of the mooring line, given by

$$\text{Sway}2\text{: }F_{\text{sway}}^{\text{vertical}}\left(t\right)=|F_{\text{sway}2}|\cdot \text{sign}\left(\sin\left(\frac{2\pi }{T_{\text{timer}}}t\right)\right),$$ (29)

where $\left|F_{\text{sway}2}\right|$ is a tunable constant force amplitude, typical between 10%–40% of max sway thrust; and $T_{\text{timer}}$ is a timer or a switching period to change the direction of the sway controlled force. In the sea trial (Section 7.3; Arntzen (2024)), $T_{\text{timer}}=60\;s$ . The control law in Equation 29 will create a “yo-yo” sway motion, combined with constant descent, produces a spiral scan around the line.

For the yaw controller law, the goal is to keep the mooring line midpoint in the middle of the captured frame.

This is given by

$$\text{Yaw: }M_{\text{yaw}}^{\text{vertical}}=k_{\text{yaw}}\cdot x_{\text{norm}},$$ (30)

where $k_{\text{yaw}}$ is the proportional gain to keep the mooring line in the middle of the captured frame.

The heave controller law adjusts the descent rate based on the inclination angle θ , according to

$$\text{Heave: }F_{\text{heave}}^{\text{vetical}}=k_{\text{heave}}\cdot d\cdot \cos|\theta |,$$ (31)

where $k_{\text{heave}}$ is a constant to scale the heave control force; d∈{−1,1} is a user input for vertical direction (1 for descent and −1 for ascent), and θ is the inclination angle of the mooring line ( θ=0° for a vertical line and θ=90° for a horizontal line).

#### 6.3.2 Vertical to horizontal

When the line becomes more horizontal (determined by depth or inclination threshold), the drone switches to a transition control mode. The objective is to align the drone horizontally with the mooring line. The controller law is detailed as below.

The surge controller law is the same as in Vertical mode (Equation 27). The sway control force is kept to a constant value, moving the drone lengthwise along the mooring line, given by

$$\text{Sway: }F_{\text{sway}}^{\text{transition}}=\text{constant}.$$ (32)

The yaw controller is the same as in Vertical mode (Equation 30). The control force in heave is set to 0 such that it does not collide with the seafloor, given by.

$$\text{Heave: }F_{\text{heave}}^{\text{transition}}=0.$$ (33)

#### 6.3.3 Horizontal inspection

As the observed mooring line angle passes a threshold for transition mode, the controller is switched to the last part, i.e., the horizontal section. The goal is to wandering around the mooring line lying on the seabed. The control strategy in surge and sway is same as the transition “Vertical to Horizontal” mode.

The yaw controller goal is to wander around the mooring line lying on the seabed, according to

$$\text{Yaw: }M_{\text{yaw}}^{\text{horizon}}=|M_{\text{yaw}}|\cdot \text{sign}\left(\sin\left(\frac{2\pi }{T_{\text{timer}}}t\right)\right),$$ (34)

where $\left|M_{\text{yaw}}\right|$ is a tunable constant moment amplitude, with similar tuning strategy as in Equation 29; and $T_{\text{timer}}$ is a timer, same as in Equation 29. The heave is regulated such that the mooring line is in the bottom quarter of the camera frame, given by

$$\text{Heave: }F_{\text{heave}}^{\text{horizon}}=k_{\text{heave}}\cdot \left(y_{\text{norm}}-y_{\text{desired}}\right),$$ (35)

where $y_{\text{norm}}\in \left[-1,1\right]$ is the normalised vertical offset of the line midpoint in the captured frame; and $y_{\text{desired}}$ is the desired vertical offset of the midpoint in the captured frame and is set to bottom quarter of the frame.

An illustration of the inspection path along the mooring line is shown in the right hand side of Figure 10 where the Sway2 strategy is visualised.

The resulting control vector is given by

$$F_{c}={\left[\begin{array}{cccc} F_{\text{surge}} & F_{\text{sway}} & F_{\text{heave}} & M_{\text{yaw}} \end{array}\right]}^{T},$$ (36)

The control vector (Equation 36) is normalised and then sent to the drone using the Blueye SDK. Each axis includes a tunable gain that can be adjusted in real time via the GUI. The thrust allocation and thruster control will be done inside the drone’s software and will not be presented here.

### 6.4 Implementation

The control strategy is implemented using ROS 2 due to its built-in support for real-time communication (Macenski et al., 2022). The ROS 2 node chain_controller contains most of the control logic. This node subscribes to visual measurements of the mooring line, which are published after image processing, and computes continuous commands in surge, sway, heave, and yaw. These commands are sent as DesiredVelocity messages to the/desired_velocity topic, which interfaces with the drone’s internal thrust allocation system.

## 7 Simulation and experimental results

This section presents the simulation and experimental results, along with corresponding analysis of the proposed vision-based inspection framework for mooring lines using a low-cost underwater drone. The results are structured to demonstrate the performance of individual system components, i.e., image processing, control, object detection, and mapping. Simulation trials were conducted to validate the full inspection strategy, including vertical descent, transition, and horizontal inspection, and to verify the effectiveness of the spiral path generated by the control system. These simulations complement the dock and sea trials. Key observations and limitations are discussed alongside the results, providing insights into the robustness, effectiveness, and future improvement areas of the system. An overview of the key parameters and outcomes from the simulations and experiments is provided in Table 7. Full quantitative results, detailed parameter settings, other scenario runs and raw datasets are provided in the master’s theses by Arntzen (2024); Elseth and Øvstaas (2025).

**TABLE 7:** Summary of simulation and experimental results.

Trial	Environment/Conditions	Objective	Key metrics/observation	Outcome
Simulation of full inspection	Ideal. Depth: 0 → seabed	Validate vertical descent, transition, and spiral horizontal inspection path	Spiral trajectory generation; Vertical-to-horizontal transition; No coverage gaps	Semi-helical path ensures full 360° coverage; no missed sections
Dock trials (Guidance only)	Calm water, synthetic rope. Depth: 0 → 10 m	Mooring line following	Depth tracking error ± 0.2 m; Thruster surge/sway/yaw within expected bounds; Automatic ascent at 4 m	Reliable mooring-line following; successful autonomous descent and ascent while keeping line centered
Sea trial Sway1 (31 Mar 2025)	Open sea, surface currents up to 0.25 m/s, marine snow. Depth: 10 → 68 m	Mooring line following with Sway1 strategy; real-world condition	Surge thrust oscillations 0%–75% max; Sway commands ≤25 % max; Manual CVI segment at 400–450 s	Completed autonomous inspection 10–68 m; manual CVI captured shackle video for YOLOv5 training
Sea trial spiral (Spring 2024)	Open sea, changing light conditions. Depth: 5 → 80 m	Mooring line following with Sway2 strategy; real-world condition	Downward heave + alternating sway; Continuous yaw adjustments	Spiral pattern executed as planned; reconstructed camera trajectory; and validated mapping feasibility
Dock trials (Shackle detection)	Calm water, metal shackle at rope end. Depth: 0 → 3.5 m	Object detection	YOLOv5 detection at 3.5 m; Detection flag triggered reverse command	Successful shackle detection to prompt the return, supporting completed autonomous inspection
Post-processing (ORB-SLAM3 and Meshroom)	Video from sea trials (Spring 2024). Depth: 5 → 80 m	Mooring line 3D reconstruction for change detection	Tracked ORB features highlight line structure; Meshroom yields coherent 3D mesh; RViz2 and ORB-SLAM3 visualizations consistent	3D reconstructions

### 7.1 Testing of image processing techniques

**FIGURE 11:**
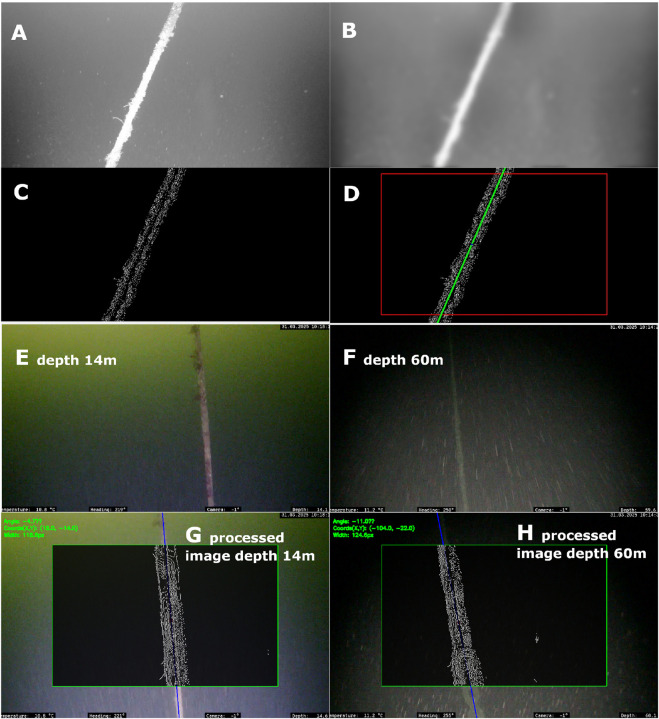
**(A)** Luminance component; **(B)** Output after guided filter and morphological operations; **(C)** Result of Canny Edge Detection; **(D)** Final image with line detected within ROI; **(E)** Frame captured near the sea surface with strong sunlight at the top of the image, and its corresponding final result **(G)**; **(F)** Frame captured near the seabed illuminated only by onboard LED with strong light at the bottom, and its corresponding final process image **(H)**.

The image processing pipeline was evaluated using recorded video data from previous inspection campaigns (Arntzen, 2024; Elseth and Øvstaas, 2025) to assess its robustness under varying lighting conditions and the presence of marine snow. Figure 11 illustrates the processing results. Subfigures A–D demonstrate the effective removal of marine snow through the successive filtering stages, while subfigures E–H show consistent detection of the mooring line across diverse lighting scenarios. The following video shows the successful detection of mooring line under a marine snow condition Image Processing Results.

### 7.2 Dock trials

Dock trials at Trondheim Biological Station (TBS) were carried out to test the drone’s capability to autonomously follow a mooring line. A mooring line was constructed by sinking a synthetic rope with a mass at the end. The water depth outside the dock is roughly 10 m allowing for testing in a controlled and calm weather condition.

These dock trials focused on guidance system including mooring line detection and control execution levels. Gain and filtration parameters were iteratively tuned to optimise the drone’s ability to track the mooring line. The path following algorithm was also tested from several angles of the mooring line to ensure robustness and stability under varying conditions. The angle of the mooring line in the camera frame is dependent on the relative heading of the drone with respect to the mooring line. The relative heading will affect how the drone’s control system operates.

**FIGURE 12:**
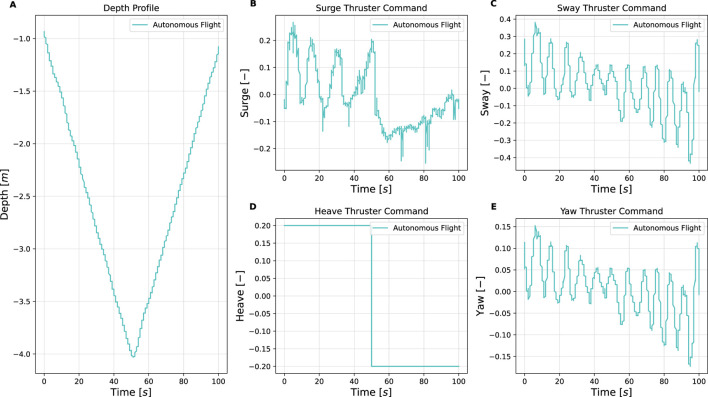
Depth profile **(A)** and thruster commands **(B–E)** for initial dock testing (Elseth and Øvstaas, 2025).

Following, a simple algorithm for automatic ascending when reaching the bottom of the rope was implemented and evaluated. Figure 12 shows the drone’s depth over time with a desired depth of 4 m. The desired depth is set ahead of the dive, and indicates the depth the drone initiates its ascent while still autonomously following the mooring line. This Figure shows that the drone can descending and ascending autonomously while controlling the surge force to keep a distance to the mooring line and controlling sway and yaw to keep the mooring line in the middle of the captured frame.

### 7.3 Sea trial

A sea trial was conducted on the 31st of March 2025. The purpose of this sea trial is to show the Sway1 control strategy in which one side of the mooring line was inspected. The weather during this sea trial was challenging. Surface currents reached approximately 0.25 m/s, exceeding the thrust capacity of the drone, especially when exposed to beam currents, where the effective cross-sectional area is greatest. To bypass this, the autonomous inspection was initiated at a depth of 10 m, where current speeds were lower. In addition to strong currents, poor underwater visibility due to marine snow posed another major challenge.

**FIGURE 13:**
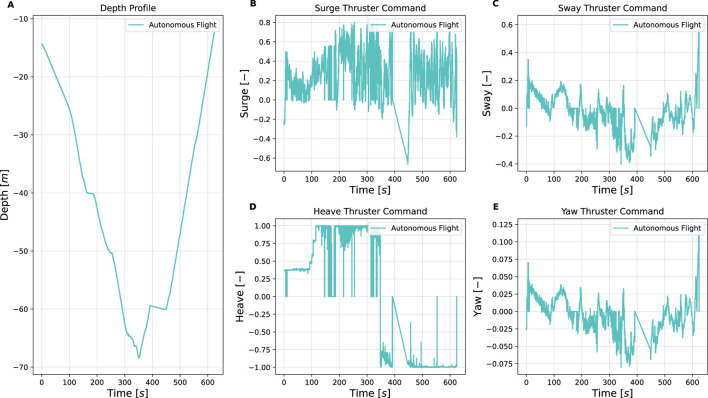
Depth profile **(A)** and thruster commands **(B–E)** during sea trial (Elseth and Øvstaas, 2025).

The autonomous inspection of the mooring line was completed successfully from 10 to 68 m depth (Figure 13). At the latter depth, the drone was operated manually to conduct a CVI to capture video data of the structural integrity of the shackle. Gathering video of the shackle was deemed important as the data can be used for further training and improvement of the accuracy and robustness of the YOLOv5 object detection model. The manual CVI can be observed as the missing data found around 400–450 s in Figure 13.

The nominal commanded thrust in surge exhibited significant oscillations and had an output range of 0%–75% of the thruster’s maximum output. The sway commands are confined within 25% of the maximum thruster output. During this trial, the thruster commands in heave were rather high due to an almost vertical mooring line. A full video of this sea trial is shown here: Sea Trial.

**FIGURE 14:**
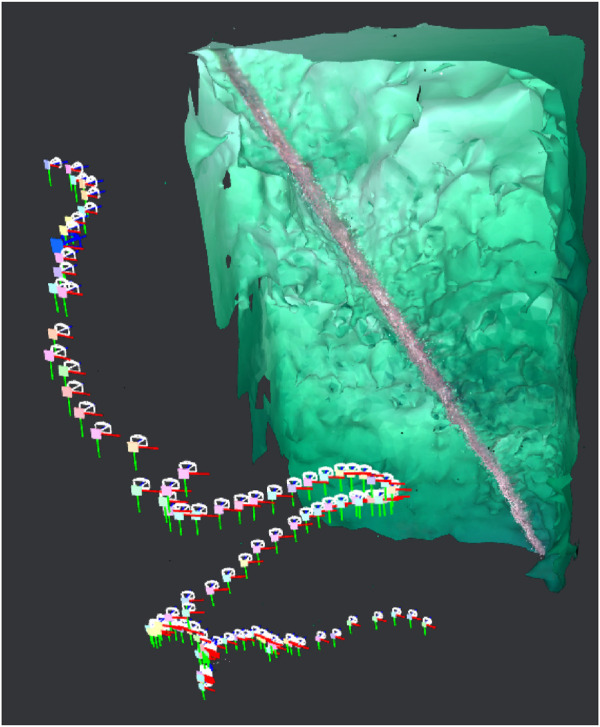
Point cloud and camera motion reconstructed from inspection video (Arntzen, 2024).

A sea trial was conducted in spring 2024 (Arntzen, 2024), to demonstrate the Sway2 control strategy with inspection spiral pattern. The results, shown in Figure 14 show the estimated camera trajectory and the resulting point cloud reconstruction of the observed environment. The trajectory reveals that the drone performed a downward heave motion while executing a spiral inspection pattern—alternating sway from side to side and continuously adjusting its yaw to keep the mooring line centred in the camera frame. This coordinated movement enabled comprehensive visual coverage of the mooring line under real-world conditions.

### 7.4 Dock trials with shackle detection

To verify the YOLOv5 object detection model in a real environment, the Blueye X3 was tested at the TBS dock with a similar setup as in Section 7.2. For the testing, a rope with a metal shackle attached to its end was lowered into the water column solely for the purpose of testing if the object detection model was capable of detecting a shackle underwater.

**FIGURE 15:**
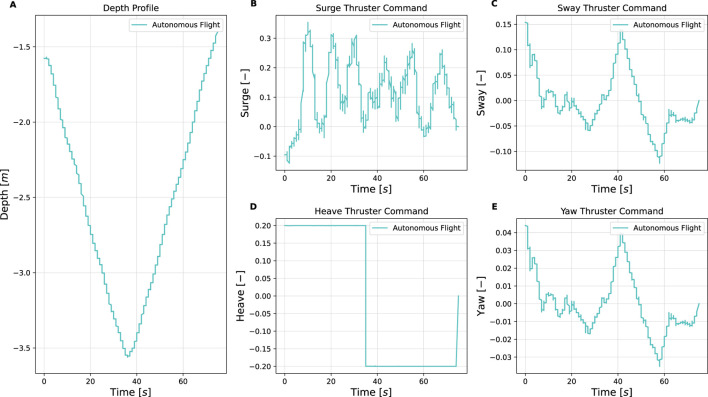
Depth profile **(A)** and thruster commands **(B–E)** for shackle detection testing (Elseth and Øvstaas, 2025).

After training the YOLOv5 model on images of shackles, the detection pipeline was integrated into the onboard perception node as described in Section 5.2. The shackle used in this testing was not part of the dataset used to train the object detection model. During the test, the live camera feed was processed through the YOLOv5 object detection model which successfully detected the shackle at around 3.5 m depth. The detection triggered a flag in the system logic, which was then published to the/reverse_command topic. The operator was then prompted to decide on the drone’s further mission. In the case of this trial, the drone was prompted to continue inspection while ascending to the surface (Figure 15). This resulted in a successful autonomous inspection along the entire makeshift mooring line. A full video of this test is shown here: Object Detection Model.

### 7.5 Simulation results

The proposed control strategy was validated through simulation to demonstrate the drone’s ability to perform full-length mooring line inspection—from vertical descent, through the vertical-to-horizontal transition, to horizontal exploration. The Sway2 strategy was applied to generate a semi-helical path around the mooring line during vertical inspection, ensuring comprehensive visual coverage.

**FIGURE 16:**
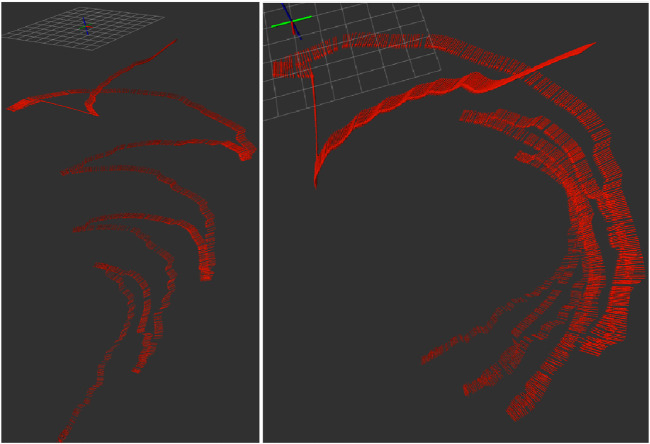
Simulated drone trajectory during full-length mooring line inspection. Side view (left) illustrates the descent and transition to horizontal inspection; and top view (right) shows the spiral pattern around the mooring line.

Figure 16 shows the simulated drone trajectory during inspection. The side view provides an overview of the full flight path along the mooring line, including the transition to horizontal motion near the seabed. The chosen descent speed is tuned to ensure no sections of the mooring line are skipped during the inspection. The top view illustrates the alternating sway pattern that produces the spiral motion, highlighting the changing circumference angle around the mooring line. Further details of the simulation setup and parameters can be found in Arntzen (2024).

### 7.6 Post processing

**FIGURE 17:**
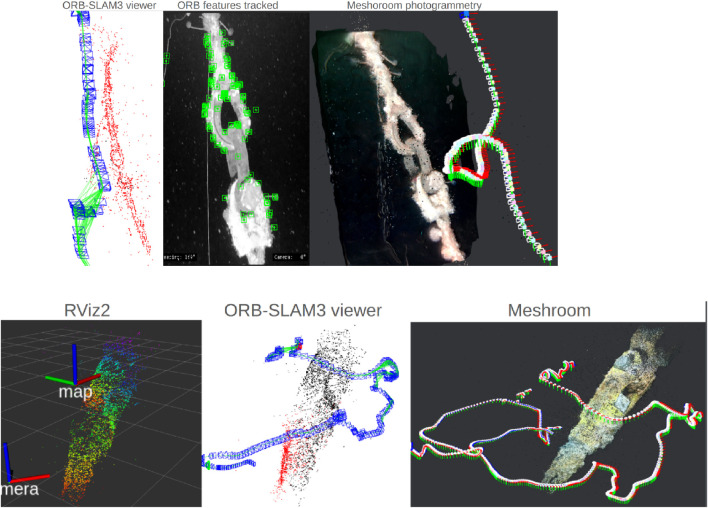
3D Reconstruction using ORB-SLAM3, tracked features and Meshroom photogrammetry (top) together with map visualization in RViz2, ORB-SLAM3 viewer, and Meshroom (Arntzen, 2024).

To explore mapping from the inspection footage, both ORB-SLAM3 and Meshroom were tested on video data from the sea trial conducted by Arntzen (2024). The first row of Figure 17 illustrates the ORB-SLAM3 viewer (left), tracked features, and the resulting 3D reconstruction from Meshroom (right). The tracked ORB features clearly highlight the structure of the mooring line. The second row of Figure 17 compares map outputs in RViz2 (left), ORB-SLAM3 viewer (middle), and Meshroom (right). These illustrate how the different tools visualise spatial information from the same video source.

## 8 Discussion

### 8.1 Summary of simulation and experimental results

In this subsection, the most important findings from both simulations and field experiments are summarised into a single, easy-to-read table. Table 7 summarizes each trial’s operating environment, depth range, key performance metrics, and overall outcome. This table provides a clear and side-by-side comparison of how the proposed vision-based inspection framework performs under ideal conditions, controlled dock tests, and challenging open-sea deployments.

### 8.2 Robustness of image processing

The image processing techniques developed in this paper showed an enhancement of the drone’s ability to autonomously inspect the mooring line. The image processing algorithm successfully detected the mooring line while removing noise (marine snow) and handling different illumination conditions.

A key challenge highlighted in Arntzen (2024) was the need for manual adjustment of filtering parameters depending on depth, due to varying lighting conditions. Near the surface, sunlight contributed to the illumination, while deeper depths relied solely on artificial lighting from the onboard LED. This made consistent filtering and detection difficult across a full mooring line inspection.

In the thesis of Elseth and Øvstaas (2025), the image processing pipeline (as described in Algorithm 2) was used at all depths without any parameter tuning. This adaptive pipeline, based on CLAHE and Canny Edge Detection, maintained reliable mooring line detection at both 19.3 m and 64.7 m depths (Elseth and Øvstaas, 2025). This marks a key improvement, as it eliminates the need for manual filter calibration between depth zones.

The parameter settings proved effective under the sea trial conditions at TBS; however, further tuning may be required when deploying the system in different environments with new visual challenges. While this robustness is promising, further investigation is needed to evaluate the limits of the pipeline under more extreme conditions—such as heavy marine snow or nighttime inspections. Future work could also explore dynamically tuned parameters or the inclusion of depth-aware filtering logic to further increase performance.

### 8.3 Path following algorithm and control system

The path following algorithm developed in this paper demonstrates that a simple, camera-based control strategy can support autonomous mooring line inspection using a low-cost drone. The results from both TBS dock trial and the sea trials confirmed that the proportional control strategy was sufficient to maintain alignment with the mooring line across 4-DOF.

The simulation validates the full inspection concept to check whether the framework works before the sea trials with uncontrolled factors. The simulations demonstrated that the drone could successfully follow the entire mooring line, transitioning from vertical to horizontal inspection while maintaining coverage along the full length. The spiral path generated by the Sway2 strategy was clearly visible in the simulated trajectory and ensured circumferential coverage of the mooring line during descent. However, the simulation were conducted in idea conditions where no environmental effect was accounted for.

In contrast, the sea trials were constrained by time and weather conditions, which prevented a full-length mooring line inspection. The sea trials primarily demonstrated the system’s ability to descend towards the shackle and ascend back to the surface, rather than executing a complete vertical-to-horizontal inspection sequence. Nonetheless, the system maintained alignment with the mooring line during these partial inspections, even under suboptimal visibility and current conditions.

For Sway1 control strategy demonstration in the 2025 sea trial (Elseth and Øvstaas, 2025), the drone was tested from several angles to evaluate operability under different perspectives of the mooring line. The sideways tracking of an angled mooring line provided the most stable performance due to consistent distance to the mooring line. However, front- and back-facing tracking introduced challenges related to the catenary shape of the rope. From this angle, more aggressive gains for surge and heave were needed, as the mooring line was constantly moving toward or away from the drone.

For Sway2 control strategy showed particular promise in simulation and the 2024 sea trial (Arntzen, 2024). However, future development could focus on enhancing the controller by incorporating the drone’s heading to ensure more robust and consistent 360-degree coverage of the mooring line in complex and dynamic underwater environments.

The implementation of a fallback state when sight of the mooring line was lost reduced downtime, and allowed the drone to re-orient itself toward the mooring line and continue the inspection. This proved useful in situations with rougher environmental conditions.

Despite successful trials, several limitations were observed. The control system relies entirely on visual feedback and does not incorporate inertial or velocity data in its control loop. This makes it more sensitive to disturbances and susceptible to drift over time. The system would likely benefit from a SLAM-based setup, which could enable loop closure if the drone revisits previously observed points along the mooring line. However, due to limited onboard computing resources and battery capacity, integration of such a solution proved challenging. An alternative approach could be to enhance the sensor suite with a Doppler Velocity Log (DVL) or sonar. However, these sensors are generally expensive and would increase the overall cost of the system.

Another limitation lies in the fixed gain structure. Although real-time tuning is possible through the GUI, it requires human intervention, limiting the overall LoA. To increase the LoA, an adaptive gain scheduling approach could be introduced, where gains are dynamically adjusted based on input data such as depth, drone orientation, or the confidence level in object detection.

Although the proposed proportional, vision-based controller proved effective in both simulation and partial sea trials, it does not explicitly account for the highly coupled nonlinear dynamics of the drone, nor for model uncertainties, input constraints, and time-varying ocean currents. In practice, currents can introduce drift and lateral forces on the drone, while the thruster constraints and vehicle dynamics exhibit nonlinear behavior under varying loads. Without an explicit dynamic model or disturbance observer, the controller treats these effects as unstructured disturbances, relying solely on reactive visual feedback. As a result, the system’s performance may degrade in stronger or rapidly changing current conditions, and actuator saturations or control limits may be reached unexpectedly.

### 8.4 Cost-efficiency of low-cost drone-based inspection

The proposed inspection framework leverages a compact, commercially available underwater drone integrated with low-cost onboard computing and vision hardware. This setup significantly reduces operational expenses compared to traditional ROV-based inspections (Ford et al., 2020), which require high-end equipment, support vessels, and specialised personnel, and are often constrained by weather and logistics.

The proposed system, including the drone platform (e.g., Blueye X3), Raspberry Pi-based edge AI processing, and vision models, costs under €24,000 and can be operated by a single technician from a Remote Operation Centre (ROC). Such a configuration is particularly advantageous for recurring inspections in offshore aquaculture or renewable energy contexts, where it enables frequent, flexible, and cost-effective monitoring.

Table 8 provides a comparative overview of the key cost elements. Traditional inspection methods may incur per-inspection costs of up to €200,000 due to vessel rental, crew, and equipment, and are typically performed once or twice per year. In contrast, the proposed solution allows for inspections on-demand at a fraction of the cost (approx. €20,000 per inspection), with additional benefits in mobility, data availability, and reduced downtime.

**TABLE 8:** Estimated cost comparison–traditional vs. proposed inspection approach.

Cost component	Traditional inspection (ROV-based)*	Proposed framework (low-cost drone + edge AI)**
Infrastructure	Mother vessel €10,000–€30,000 per day	Possible docking station €100,000 per whole life cycle
Hardware	ROV €5,000 per day rental	Low-cost drone €24,000 (Blueye X3)
Crew (ROV + vessel)	4–6 personnel (€4,000–€6,000 per day)	1 personnel for the whole fleet of drones (€1,000 per day)
Other inspection equipment	Depends on the type of inspection €1,000-€10,000	Maintenance cost €1,000
Total estimated cost per inspection	€200,000	€20,000
Other costs, not included in the estimates		
Inspection frequency	1–2 times per year	10 times with the same budget
Mobility and flexibility	Limited by mobilisation needs and weather conditions	High, easy deployment, less dependent on surface weather
Data post-processing	Offline, often manual review by human	Autonomous change detection
Downtime Impact	High due to scheduling delays	Low, rapid redeployment possible

^*^ based on Ford et al. (2020), 2020 costs.

^**^ approximate estimates based on typical operational conditions and available market data as of 2025. There might be unseen costs. The comparison is intended to highlight relative differences in scale rather than serve as an exact financial projection.

### 8.5 Real-time processing performance on edge hardware

The inference pipeline, including object detection with YOLOv5s and basic image enhancement, was executed onboard a Raspberry Pi during test missions. CPU usage was monitored continuously, with observed utilisation remaining below 100% during the detection and tracking processes. The drone performed its control actions and feedback loops without delays, freezes, or reboots, indicating that the real-time computational demands were well within the system’s capabilities. This confirms that the proposed vision-based inspection framework is suitable for edge deployment on resource-constrained hardware, a key requirement for scalable, autonomous underwater inspection.

### 8.6 Manual intervention during shackle identification

Upon detecting a shackle, the system enters a hold state where operator input is needed. A GUI window, as illustrated in Figure 7, allows the user to confirm whether the drone should initiate its ascent or continue the inspection while descending.

This approach would enable the drone to use its perception of the environment or site-specific knowledge for autonomous decision-making upon detecting a shackle or other structural component on the mooring line. For example, if the drone determines—based on acoustic data—that it is near the seabed and simultaneously detects a shackle, it could autonomously decide to perform a CVI of the shackle before initiating its ascent along a different angle of the mooring line, without requiring human intervention. Ascending with a different heading to the mooring line would also ensure an exhaustive inspection and provide a more comprehensive visual dataset, which is likely to improve the accuracy and robustness of 3D reconstruction by capturing the structure from multiple angles.

### 8.7 3D reconstruction and mapping

The post-processing comparison shows that ORB-SLAM3 is able to track local image features and generate a sparse 3D map in real time. However, the resulting point cloud in RViz2 becomes significantly noisier. This is likely due to repeated false detections caused by marine snow, which accumulates in OctoMap and introduces large clusters of outliers.

Another challenge lies in the structure of the mooring line itself. Its repetitive structure and narrow viewing angle from the drone makes loop closure difficult, causing drift to accumulate over time. One of the fundamentals in SLAM is recognising previously visited areas for drift correction, this limits its mapping accuracy in this case.

On the other hand, Meshroom performs global optimisation offline. It uses a scale-invariant feature transform (SIFT) for feature detection, resulting in denser reconstructions and lower drift. This may make it better suited for a detailed post processing analysis. However this method is not applicable for real-time operations due to the limited computational power onboard the drone.

## 9 Conclusion and future work

This study demonstrated a cost-effective and autonomous solution for mooring line inspection using the Blueye X3 drone. A modular ROS 2 based architecture was implemented, combining real-time image processing, a YOLOv5-based object detection module, and a simple controller for mooring line following. The image processing pipeline, including Guided Filtering, CLAHE, Morphological Transformation, and Canny Edge Detection, handled challenges such as marine snow and varying illumination. The extracted visual features were used to guide the drone’s motion across four degrees of freedom (surge, sway, heave, and yaw) through a proportional controller.

The system was validated through simulation, video replay testing, and sea trials, demonstrating that cost-effective, off-the-shelf observation-class underwater drones can reliably perform mooring line monitoring and inspection when equipped with suitable visual perception and control strategies. Furthermore, the acquired visual data were successfully used for 3D reconstruction of the mooring line via tools like ORB-SLAM3 and Meshroom, supporting the potential for future change detection and defect identification.

This study contributes:

• A modular and scalable system architecture compatible with low-cost underwater platforms.

• The first demonstration of autonomous inspection using a cost-effective Blueye X3 in real sea conditions.

• A validated pathway to embed AI-based perception and control within resource-constrained robotic platforms.

While current limitations include the need for parameter tuning and the absence of inertial feedback, this work provides a strong foundation for affordable and scalable autonomous underwater inspection, with clear pathways for enhancement through sensor fusion and adaptive control.

### 9.1 Proposed future work

There are several promising directions for extending the current system. First, the fixed-gain proportional controller used in this work could be replaced by a more advanced control strategy, such as a full PID controller or a model leveraging adaptive gain scheduling. These approaches may reduce oscillations and improve responsiveness, particularly during extended inspection missions.

To improve robustness and situational awareness, the integration of additional sensors should be explored. For example, a Doppler Velocity Log (DVL) could help reduce positional drift and improve the reliability of visual path-following, as DVLs are unaffected by marine snow or fluctuations in lighting. However, the inclusion of such sensors introduces economic and payload constraints. For instance, the Water Linked DVL for the Blueye X3 is priced at $7,890 (WaterLinked, 2024)—which may limit its applicability in cost-sensitive deployments.

Another key area of development is the implementation of fully autonomous decision logic for shackle detection and corresponding maneuvers. Automating this process would eliminate the need for operator input, thus raising the LoA and mission continuity.

Moreover, research into real-time VSLAM techniques on constrained platforms could significantly expand the system’s capabilities. Simplified or event-based loop closure methods should be explored to enable ORB-SLAM3 or equivalent systems to run efficiently on limited onboard hardware, such as the embedded processors on the Blueye X3 (Arntzen, 2024). This may include testing whether place recognition can be achieved through mission-specific keyframes or distinct visual features such as shackles.

Future work will expand the simulation experiments to include varied environmental and failure scenarios and report quantitative metrics for selected control and mapping approaches, including detection precision/recall, runtime, localisation accuracy, map completeness, and mission success rate. These results will enable sensitivity analyses and statistical evaluation of system robustness.

Bioinspired control and optimisation methods have shown promise in managing actuator faults and navigating in uncertain conditions (Tutsoy et al., 2024). These techniques could be adapted for underwater drone inspection systems to enable more resilient and efficient motion planning in the presence of vehicle nonlinear dynamics, mooring line dynamics and unknown external disturbances.

Finally, future work will focus on a systematic benchmarking of various onboard processing units against the demanding requirements of real-time VSLAM tasks within the underwater domain. This is important to optimise the trade-offs between image processing fidelity, energy consumption, and mission endurance. Recent advancements in embedded processing, e.g., NVIDIA Jetson series (NVIDIA, 2023), which offer capabilities for real-time inference and embedded AI acceleration while maintaining manageable power envelopes, show a promise for accelerating the practical integration of advanced visual perception directly onboard the underwater drone.

## Data Availability

The datasets presented in this study can be found in online repositories. The names of the repository/repositories and accession number(s) can be found below: https://www.youtube.com/watch?v=RRCnPM2YfqU&list=PLm_K53dPd9QsbjtfPZmFla-n3JSzyvZGy&index=1&ab_channel=JakobRude%C3%98vstaas.
